# Toward Monodomain Nematic Liquid Crystal Elastomers of Arbitrary Thickness through PET-RAFT Polymerization

**DOI:** 10.1021/acs.macromol.4c00245

**Published:** 2024-05-28

**Authors:** Stuart R. Berrow, Richard J. Mandle, Thomas Raistrick, Matthew Reynolds, Helen F. Gleeson

**Affiliations:** † School of Physics and Astronomy, University of Leeds, Leeds LS2 9JT, U.K.; ‡ School of Chemistry, University of Leeds, Leeds LS2 9JT, U.K.

## Abstract

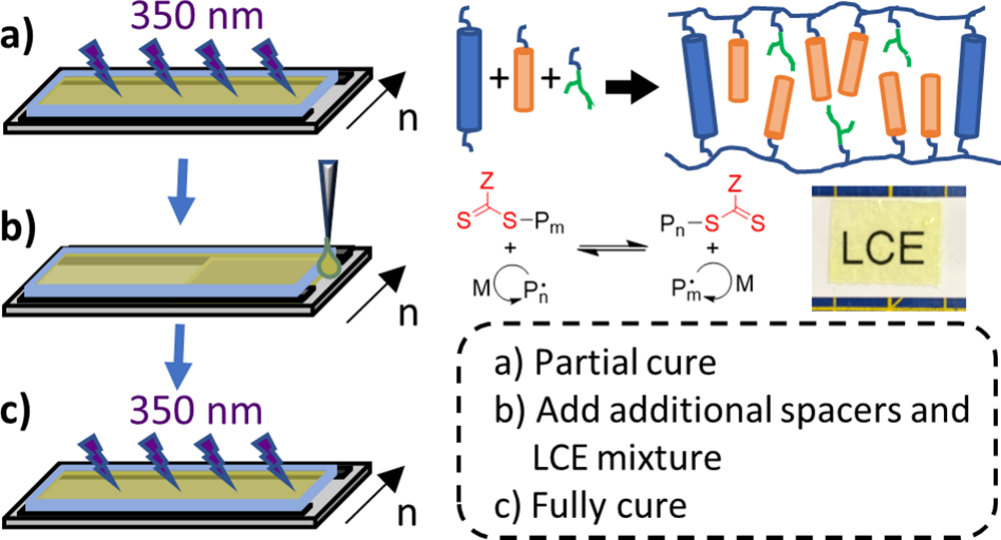

Liquid crystal elastomers (LCEs) are polymeric materials that are proposed for a range of applications. However, to reach their full potential, it is desirable to have as much flexibility as possible in terms of the sample dimensions, while maintaining well-defined alignment. In this work, photoinduced electron/energy transfer reversible addition–fragmentation chain transfer (PET-RAFT) polymerization is applied to the synthesis of LCEs for the first time. An initial LCE layer (∼100 μm thickness) is partially cured before a second layer of the precursor mixture is added. The curing reaction is then resumed and is observed by FTIR to complete within 15 min of irradiation, yielding samples of increased thickness. Monodomain samples that exhibit an auxetic response and are of thickness 250–300 μm are consistently achieved. All samples are characterized thermally, mechanically, and in terms of their order parameters. The LCEs have physical properties comparable to those of analogous LCEs produced via free-radical polymerization.

## Introduction

Elastomers are lightly cross-linked polymers, capable of large deformations when subject to stress.^1^ Liquid crystal elastomers (LCEs) combine the properties of elastomers and liquid crystals by incorporating mesogenic units into the polymer structure, either directly into the polymer backbone (main chain LCEs) or via a flexible spacer (side chain LCEs).^2^ In LCEs, the polymer backbone conformation and the self-organizational properties of liquid crystal mesogens are coupled. This results in LCEs being able to undergo reversible shape changes when subject to a stimulus that results in a phase transition, with commonly exploited stimuli being temperature or irradiation.^2^ The magnitude of these shape changes has been shown to be comparable to animal muscles.^3,4^ This shape-changing ability has led to LCEs being investigated for applications such as actuators, shape-memory materials, artificial muscles, and soft robotics.^3−9^ More recently, some nematic LCEs, including those used as exemplars in this work, have been reported to display auxetic behavior, further expanding the scope of their potential applications.^10,11^

In order to maximize the magnitude of a given behavior, such as the shape memory effect or auxetic behavior, high-quality, well-specified alignment of the mesogens is required.^12^ In many cases, monodomain alignment, i.e., macroscopic alignment of the mesogens in a single direction throughout a bulk sample, is essential. However, without an external surface, electric or magnetic field to align the mesogens within an elastomer, polydomain samples, which consist of areas of localized, randomly oriented nematic order, submicron in size, are obtained.^13^

This work reports an approach to produce uniformly aligned nematic liquid crystal elastomer films of arbitrary thicknesses using PET-RAFT polymerization. This approach attempts to overcome the limitations of conventional alignment methods in the production of LCEs of increased thickness. These limitations vary depending on the method of alignment attempted. For example, the first case in which monodomain alignment was achieved in an LCE was reported by Kupfer and Finkelmann in 1991.^12^ Their method consists of partial curing of a polydomain LCE, followed by the application of uniaxial strain resulting in monodomain alignment.^12,14^ The cure is then completed while the LCE is under strain to yield a monodomain sample.^15−18^ However, this methodology currently relies on LCEs containing functional groups of differing reactivity, so is not applicable in all systems.^19^ However, the advance of dynamic covalent chemistries may present an opportunity to overcome this limitation.^13,16,20^ Additionally, a two-stage approach similar to that of Kupfer and Finkelmann utilizing both thermal and photochemical reactions to produce mechanically aligned monodomain LCEs has shown success and enabled complex alignment patterns to be produced.^21^

Since the original work of Kupfer and Finkelmann, other methods have been developed to align LCEs, often replicating alignment methods applied to low molecular weight liquid crystals. Such methods include the application of electric/magnetic fields to the system to induce the desired alignment.^11,19^ While field alignment presents the advantage of no sample thickness limitations, its limitations include possible breakdown of samples, limited availability of appropriate field sources, and, in the case of electric fields, the possible alignment achievable being limited by the dielectric anisotropy of the sample.^11,19,22,23^ A relatively recent development in LCE production is the use of additive manufacturing techniques (such as 3D printing).^24−27^ These techniques present exciting opportunities for the future of LCEs, as samples with complex structures and shapes can be produced in a cost-effective manner. However, additive manufacturing too can have drawbacks depending on the method employed, including slow print speeds, low resolution, and moderate alignment quality.^24^

A popular method to achieve monodomain LCEs is the use of surface alignment, where the mesogens align topologically with a uniaxially rubbed polyimide or poly(vinyl alcohol) layer, or a photoalignment layer with a more complex alignment pattern.^3,19,28^ This approach can enable complex and unique director patterns to be achieved, which can result in the LCEs exhibiting some interesting properties.^28−30^ However, surface alignment is typically limited to sample thicknesses of up to, at most 100 μm. This is because while the alignment is enforced at the rubbed substrates, there is an interaction length over which the alignment preference is maintained.^19^ The factors affecting this interaction length are complex and include the strength of intermolecular interactions and the affinity of the material for a given substrate (anchoring energy).^19^

Such limited sample thickness can present issues, for example, limiting the forces the LCE is capable of imparting, or indeed the magnitude of interesting phenomena such as the auxetic response.^3,13,19,28^ This can, in turn, diminish the applicability of LCEs for some of their proposed applications as actuators or, in the case of auxetic LCEs, shock-absorbing or delamination-resistant materials. Therefore, irrespective of the desired application, there is interest from the LCE community in approaches that achieve high-quality LCE samples of increased thickness.

Recent work from Guin et al. reports the production of laminate LCEs to overcome thickness limitations, and subsequent work from McCracken et al. employed the same method to produce layered LCEs of up to 1 mm thickness.^28,31^ Further work from the same group applied this layering approach to combine LCEs of varying moduli, in order to produce interesting macroscopic behavior, notably leaping.^32^

Free-radical polymerization is a technique that is commonly applied in the synthesis of LCEs and is the method employed in the synthesis of the auxetic LCEs reported previously within our group.^11,14,33,34^ Free-radical polymerization is particularly applicable to LCEs as it can be photoinitiated, removing any requirement for elevated temperatures to initiate polymerization. This effectively allows polymerization to be undertaken at any temperature. Thus, the polymerization can be undertaken at a temperature where the liquid crystal mesophase is stable, and hence, any desired alignment imparted in the monomer nematic phase can be retained in the polymer.

**Figure 1:**
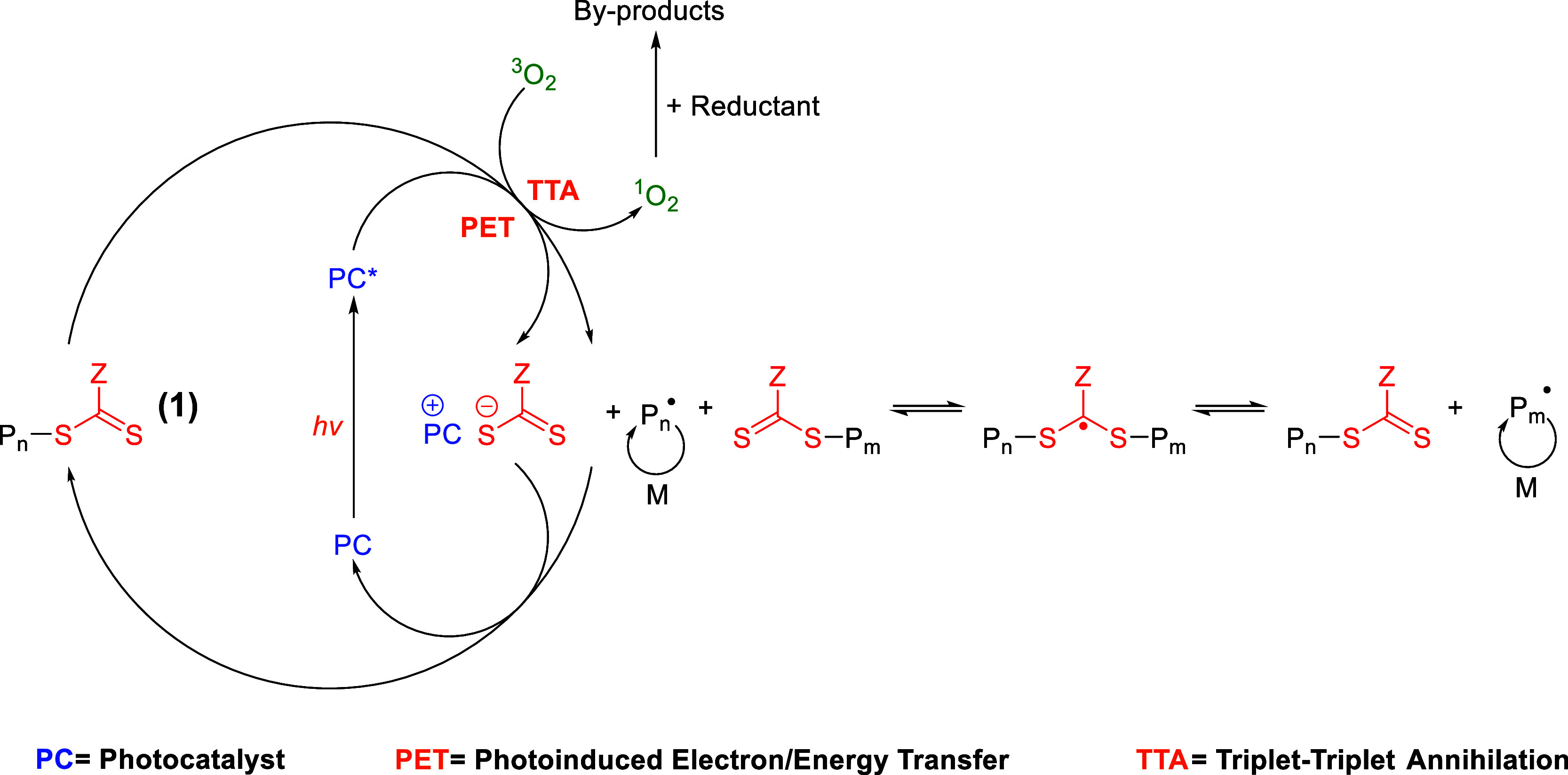
Mechanism for PET-RAFT polymerization, adapted from Phommalysack-Lovan et al.^36^

Reversible addition–fragmentation chain transfer (RAFT) polymerization is a reversible deactivation radical polymerization (RDRP) that employs thiocarbonylthio species (species (1) in Figure 1) as chain-transfer agents (a.k.a RAFT agents).^35,36^ The introduction of a thiocarbonylthio species into the polymerization allows chain termination reactions to be minimized.^35,36^ Mechanistically, RAFT removes the dependence on persistent radicals that exist in free-radical polymerization, with propagating radicals and dormant chains existing in equilibrium.^35,36^ Therefore, RAFT effectively results in living radical polymerizations and affords greater control over the polymeric products than can be achieved with free-radical polymerization.

Photoinduced RAFT has been widely described in the literature and has features that make it a suitable candidate for the synthesis of LCEs, namely, a lack of temperature dependence which allows polymerization to be conducted in a condensed phase.^37−45^ An exciting development in this area is the development of photoinduced electron/energy-transfer RAFT (PET-RAFT) polymerization. PET-RAFT combines photoredox catalysis and RAFT polymerization into a polymerization process that has some very promising characteristics.^35,36^ Upon irradiation, the photocatalyst is excited and transfers an electron (or energy) to the thiocarbonylthio moiety.^36^ This results in the production of radicals that initiate the RAFT process. Propagating radicals may then interact with the oxidized photocatalyst to effectively reset the system, meaning that the mechanism resembles a catalytic cycle unlike that of free-radical or conventional RAFT polymerization (Figure 1).^36^ One of the interesting capabilities of PET-RAFT that has been exploited in the literature is that the reaction can effectively be turned “on and off”.^37,38,42,46^ Irradiation results in the initiation of the RAFT process, and the removal of the light source resets the system, yielding dormant polymer chains. The dormant polymers could then be reinitiated to continue the reaction as desired. While such stop-start capabilities could be achieved through other chemistries, such as the thiol–ene chemistry employed by Guin et al., it would not be feasible by a conventional free-radical polymerization.^28^ Thus, using these capabilities of PET-RAFT could expand the scope of radical-based LCE production.

In this paper, we describe the utilization of the stop-start capability of PET-RAFT polymerization to tackle the sample thickness limitations of surface-aligned LCEs. In 2018, Guin et al. demonstrated the use of aligned LCEs as alignment layers for subsequent LCE layers in laminate LCEs.^28^ In this work, we build on that report, by using a partially cured elastomer sample, prepared through PET-RAFT polymerization, as an alignment substrate for subsequent elastomer production. The report from Guin et al. suggests that adding a “layer” of the uncured PET-RAFT mixture into an alignment cell consisting of one partially cured elastomer and a glass/polymer substrate will allow the new “layer” to align.^28^ Because of the stop-start capability of PET-RAFT, when the curing reaction is initiated, not only is the second layer cured, but the curing of the partially cured initial “layer” is reinitiated. This should allow the two “layers” to be chemically bound and result in a uniform, integrated elastomer film of increased thickness, offering a significant advantage over the earlier approaches.

## Experimental Section

### Materials

2-Methyl-1,4-phenylene bis(4-((6-(acryloyloxy)hexyloxy)benzoate (RM82) was obtained from Ambeed Inc. (Arlington Heights, IL, USA). 4′-Hexyloxy-(1,1′-biphenyl)-4-carbonitrile (6OCB), 2-(((Dodecylthio)carbonothioyl)thio)-2-methylpropanoic acid (DDMAT), and potassium iodide were obtained from Fluorochem Ltd. (Glossop, UK). Methylbenzoyl formate (MBF), 2-ethylhexyl acrylate (EHA), dimethylformamide (DMF), triethylamine, *n*-hexane, ethyl acetate, and poly(vinyl alcohol) (average *M*_w_ 13,000–23,000) (PVA) were obtained from Sigma-Aldrich Ltd. (Gillingham, UK). Chloroform, methanol, dichloromethane (DCM), potassium carbonate, tetrahydrofuran (THF), potassium hydrogen carbonate, sodium chloride, and acryloyl chloride were obtained from Fisher Scientific Ltd. (Loughborough, UK). 4-Cyano-4′-hydroxybiphenyl, 6-chloro-1-hexanol, and Tris(2-phenylpyridine)iridium (Ir(PPy)_3_) were obtained from Apollo Scientific Ltd. (Stockport, UK). All materials were used as obtained without further purification.

### LCE Cell Fabrication

The LCEs were synthesized in bespoke molds (cells), which were made in accordance with previous literature.^10,11,47,48^ Full information regarding the fabrication of these cells can be found in the Supporting Information.

### LCE Synthesis: Free-Radical Polymerization

The elastomers produced via free-radical polymerization were prepared according to previous literature.^10,11,47,48^ In a typical procedure, two elastomer samples were prepared. RM82 (0.059 g, 0.09 mmol), A6OCB (0.213 g, 0.61 mmol), and 6OCB (0.381 g, 1.36 mmol) were heated to 120 °C with stirring until a homogeneous isotropic phase was obtained. The mixture was then cooled to 35 °C, followed by the addition of EHA (83.0 μL, 0.40 mmol) and MBF (5.3 μL, 0.04 mmol), and the mixture was stirred for 5 min. The mixture was then filled into the alignment cells at 35 °C via pipette, before being cooled to room temperature and allowed to stand for 30 min. The samples were then cured under 350 nm (2.5 Wcm^–2^) irradiation for 2 h. After curing, the samples were removed from the alignment cells (using a small amount of methanol if necessary to aid delamination from the substrates) and left to stand in a solution of DCM:methanol (30:70) overnight, in order to remove the nonreactive 6OCB. The samples were then allowed to dry under ambient conditions for 5 h, to yield the final ∼100 μm thick elastomer films.

### LCE Synthesis: 2× Thick PET-RAFT Polymerization

**Figure 2:**
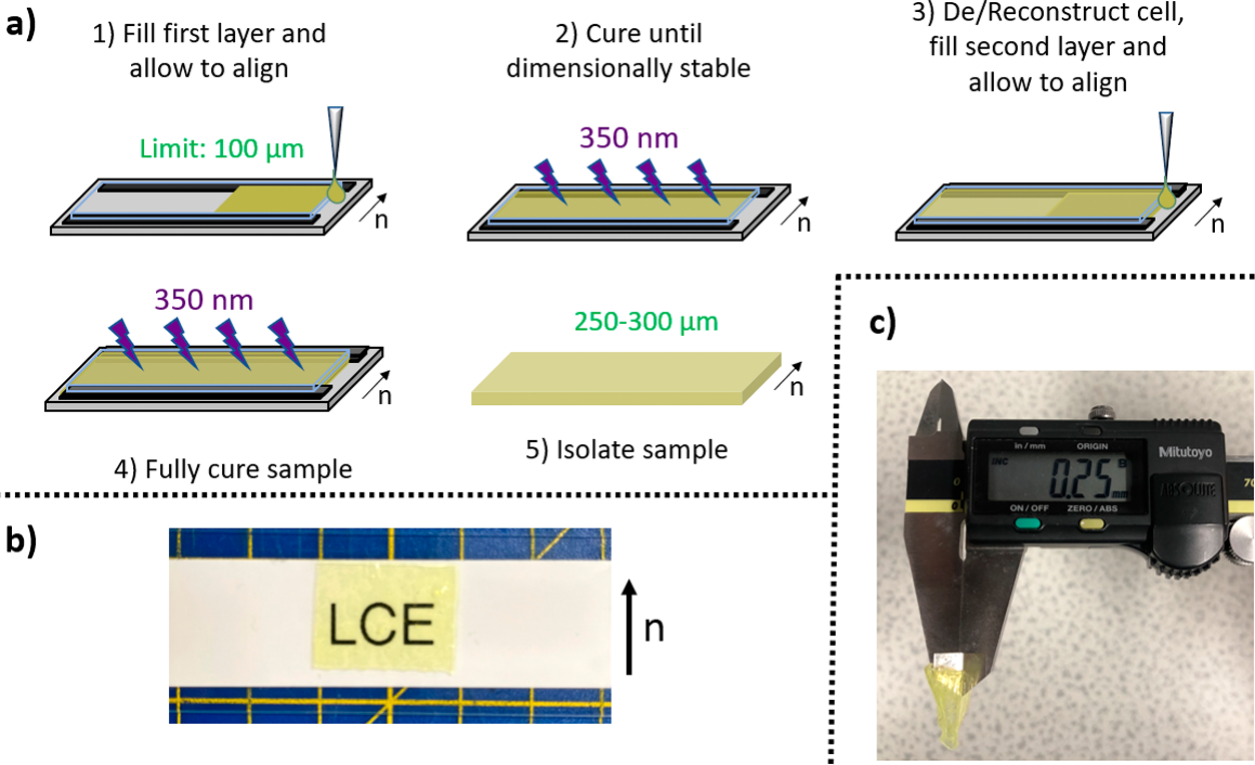
(a) Schematic representation of the elastomer production process, (b) macroscopic high optical quality of the elastomer, demonstrated by visualization of letters beneath the sample, and (c) direct measurement on the multiple-layer LCE using Vernier calipers.

Ir(PPy)_3_ (0.5 mg, 0.001 mmol), DDMAT (9.2 g, 0.025 mmol), RM82 (0.060 g, 0.089 mmol), A6OCB (0.217 g, 0.620 mmol), and 6OCB (0.388 g, 1.387 mmol) were heated to 120 °C with stirring until a homogeneous isotropic phase was obtained. The mixture was then cooled to 35 °C, followed by the addition of EHA (84.5 μL, 0.406 mmol), and the mixture was stirred for 5 min. The mixture was then filled into the alignment cell at 35 °C via pipette, before being cooled to room temperature and allowed to stand for 30 min. The samples were then cured under 350 nm (2.5 Wcm^–2^) irradiation until they were dimensionally stable (typically 10 min). The glass substrate was then removed from the alignment cell, and a further 100 μm Melinex 401 spacer was affixed atop the existing spacers using UVS-91 adhesive. A second glass substrate (rubbed with a PVA alignment layer) was then affixed atop the new spacers (again using UVS-91), and the adhesive was cured for 30 s under 350 nm irradiation (2.5 Wcm^–2^) (Figure 2). The reconstructed alignment cell was heated to 35 °C, and the remaining mixture was filled into the cell via pipette. The cell was then allowed to cool to room temperature and left to stand for 30 min. The samples were then cured under 350 nm (2.5 Wcm^–2^) irradiation for 2 h. After curing, the samples were removed from the alignment cells (using a small amount of methanol if necessary to aid delamination from the substrates) and left to stand in a solution of DCM:methanol (30:70) overnight. The samples were then allowed to dry under ambient conditions for 5 h, to yield the final ∼200 μm thick elastomer films.

## Results and Discussion

### Synthesis and Characterization of 6OCB–OH and A6OCB

The mesogenic monomer A6OCB was synthesized in a two-step process (Figure S2), adapted from the procedure reported by Hayata et al.^49^ The process consists of a Williamson ether synthesis to yield the 6OCB–OH intermediate in good yield. The 6OCB–OH is then esterified using triethylamine and acryloyl chloride to yield the acrylate monomer in reasonable yield. Structural analysis of 6OCB–OH, and its phase transition behavior is in agreement with that reported in the previous literature.^50^ Structural analysis of the A6OCB agrees with that in the previous literature, as does the phase transition behavior observed.^47,49,51^ An example of DSC thermogram and an example of the nematic Schlieren texture observed for the nematic phase for 6OCB–OH and A6OCB are displayed in Figures S7 and S8**,** respectively.

### PET-RAFT LCE Production

**Figure 3:**
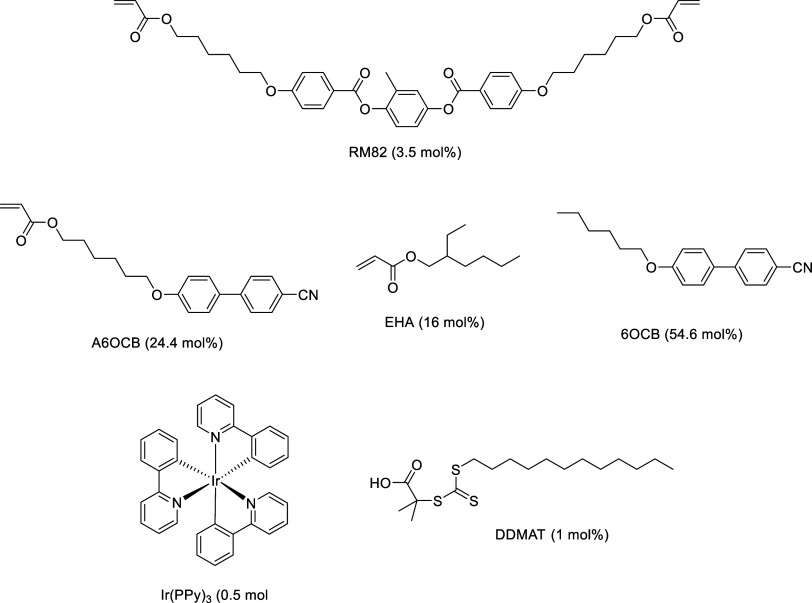
Mixture used to produce the PET-RAFT elastomers.

The elastomer formulation used in the production of the PET-RAFT elastomers is adapted from the mixture used to produce the auxetic LCE reported previously, selected for this study because of its auxetic behavior and the potential to easily produce well-aligned nematic films.^10,11^ The mixture (Figure 3) consists of the monofunctional reactive mesogen A6OCB, as well as an additional monofunctional acrylate, EHA, which imparts a plasticizing effect on the LCE. The difunctional mesogenic acrylate RM82 is employed as the cross-linker in these elastomers, and the unreactive mesogen 6OCB is used to increase the phase stability of the mixture. This 6OCB is then removed from the final elastomers by leaving the cured sample to stand in a 30:70 mixture for DCM:methanol overnight. It is of note that for the curing of the second layer of material, the sample is flipped before irradiation, to facilitate uniform curing through the sample thickness.

**Figure 4:**
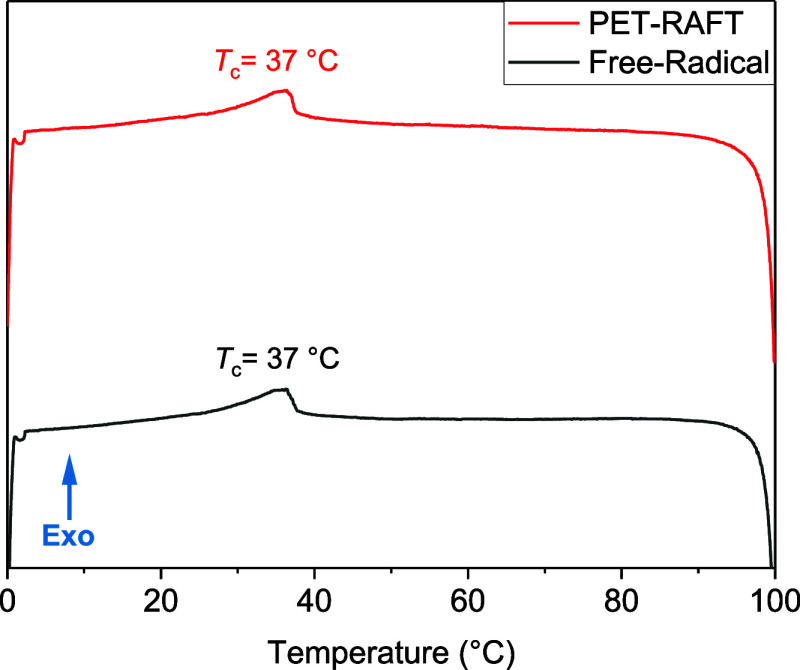
Examples of the cooling cycles obtained from the DSC thermograms of the unpolymerized elastomer precursor mixtures. The black thermogram denotes the free-radical mixture (with 1.5 mol % MBF initiator), and the red thermogram denotes the PET-RAFT mixture (0.5 mol % Ir(PPy)_3_ and 1 mol % DDMAT as “initiators”).

The free-radical initiator methylbenzoyl formate (MBF) that has been used in the previous works was replaced with Ir(PPy)_3_ and DDMAT, which serve as a photocatalyst and RAFT agent, respectively, in the PET-RAFT elastomers. This slight change in the mixture composition has no impact on the clearing temperature of the unpolymerized mixture, as evidenced by the DSC thermograms displayed in Figure 4. Both mixtures form an enantiotropic nematic phase, with a clearing point at 37 °C, in agreement with the previous literature.^10^ It is noteworthy that a small exothermic event can be observed during the polymerization of this LCE mixture (on the order of a few Kelvin). However, this is comparable to the exotherm observed during free-radical polymerization for an analogous mixture and is not significant enough to compromise the nematic phase stability. It is of course possible that if polymerization was attempted closer to the clearing temperature of a LCE mixture, some issues regarding phase stability could arise, though these are comparable to those that may occur during an analogous free-radical polymerization.

The miscibility of the Ir(PPy)_3_ photocatalyst with the other components of the mixture is sufficient to make the PET-RAFT process viable and does not present significant issues with insoluble material in the final elastomers. However, we propose that the use of an organic photocatalyst, such as eosin Y, may enable higher photocatalyst concentrations to be achieved if required. The use of Ir(PPy)_3_ also results in the elastomers exhibiting a yellow color (Figure 2c). This could present challenges if elastomers were desired for optical applications. However, elastomers are often not used in optical applications, and the issue could be avoided with alternative photocatalysts, or the removal of Ir(PPy)_3_ through washes with dichloromethane if required.

In general, the elastomers produced are of high quality. Elastomers up to 300 μm in thickness were consistently achieved, which were optically transparent and showed minimal evidence of any defects or imperfections over a large area (Figure 2c). The samples showed homogeneous thicknesses throughout, with at most a 5% variation in the sample thickness measured (Table S3), comparable to that measured for free-radical LCEs (Table S4). However, on occasion, the elastomers were isolated with some wrinkling on the surface, a consequence of the need to wash the unreactive 6OCB out of the elastomers. Immediately after curing, the elastomers are effectively swollen with 6OCB. In order to prevent crystallization of the 6OCB, the elastomers are left to stand overnight in a 70:30 mixture of methanol:DCM. At this point, the elastomers are swollen with methanol:DCM and then are allowed to dry.^52^

Experiments have also been undertaken in which the second layer added to the LCE does not contain either the Ir(PPy)_3_ photocatalyst or the DDMAT RAFT agent. The mixture used for this second layer simply consists of appropriate quantities of the monomers (RM82, A6OCB, and EHA) as well as the nematic solvent (6OCB). These experiments were undertaken to investigate the living nature of the polymerization and ensure that it was being exploited in this synthesis. The absence of the Ir(PPy)_3_ and DDMAT should mean that the second layer of material could not polymerize on its own, and thus, if the material were to be cured, it would almost certainly indicate that the first layer of material had initiated the curing of the second layer.

When these experiments were conducted, the second layer of the LCEs was seen to cure, as with the experiments previously described, where the second layer contained the photocatalyst and RAFT agent. This suggests that the living nature of the PET-RAFT mechanism is indeed exploited in these experiments. It also serves as a strong indication that the layers of the LCE are chemically bound, as the initial material initiated the polymerization within the second layer, and hence, the initial polymer network will have been extended with the new material. This would also be the case where the second layer of the material contains the photocatalyst and RAFT agent, though the additional initiating material may lead to a reduced cure time for the subsequent layer. In general, regardless of the presence of photocatalyst and RAFT agent in the second layer of the material, the LCEs behave comparably.

### LCE Curing Investigations

**Figure 5:**
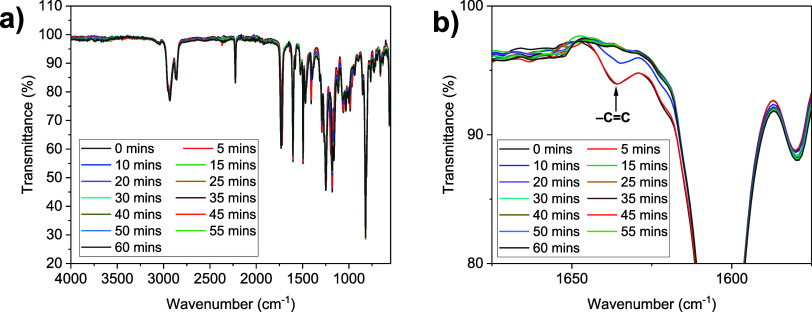
(a) Full FTIR spectra for the PET-RAFT LCE as a function of curing time and (b) region of 1575–1675 cm^–1^ for the FTIR spectra, highlighting the acrylate C=C absorbance.

Given the novel application of the PET-RAFT polymerization method to LCEs, we were interested in examining the kinetics of the curing reaction in the LCE systems studied. This was achieved via FTIR spectroscopy, as described in the experimental details in the Supporting Information. The characteristic absorption of the acrylate C=C bond at 1636 cm^–1^ was used as the major indicator of the conversion of the acrylate groups in the system, as applied to LCE conversion studies for chemistries such as thiol-Michael reactions.^52^ The full FTIR spectra (normalized to the intensity of the unreactive −C≡N stretch at 2226 cm^–1^) are displayed in Figure 5a, and Figure 5b focuses on the region of 1575–1675 cm^–1^ in order to highlight the acrylate C=C absorbance at 1636 cm^–1^.

The FTIR spectra show that upon the first 5 min of irradiation, the acrylate conversion is minimal. This is attributed to the oxygen tolerance capability afforded by the PET-RAFT mechanism. During the early stages of the irradiation, any oxygen within the system is reduced to an inactive superoxide by single electron reduction.^42^ The period for which this oxygen consumption occurs is known as an induction period, and after the induction period, the polymerization proceeds.^42^

The progression of the polymerization can be visualized in the FTIR data recorded after 10 min of irradiation, in which a significant reduction in the strength of the C=C stretch at 1636 cm^–1^ is observed. This is further evidenced by the subsequent disappearance of the 1636 cm^–1^ stretch after 15 min of irradiation. After 15 min of irradiation and for the remainder of the experiments, no further changes are seen in the stretch at 1636 cm^–1^, indicating that complete cure of the LCE is achieved within 15 min. This is further supported by the changes observed in other areas of the FTIR spectra (Figure S10), which also show a similar pattern of minimal changes for the 5 min induction period, and no further changes after 15 min. For example, the C=O stretch observed in the region of 1720–1740 cm^–1,^ which shows a shift from 1721 cm^–1^ before polymerization to 1731 cm^–1^ after complete cure is achieved. This is attributed to the change in the electron density of the carbonyl group of the acrylate, resulting from the polymerization reaction.

The FTIR data also rationalize the use of the initial cure time of 10 min for the first layer of LCE material. After 10 min, a significant portion of the material has polymerized, and the LCE on a macroscopic level is a stable, solid material. However, there is still unreacted material present to facilitate further reaction. We rationalize that the presence of a quantity of unreacted material in the initial layer of material facilitates reinitiation of the polymerization and thus enables multiple layers to be chemically bound.

### LCE Alignment and Order Parameter

**Figure 6:**
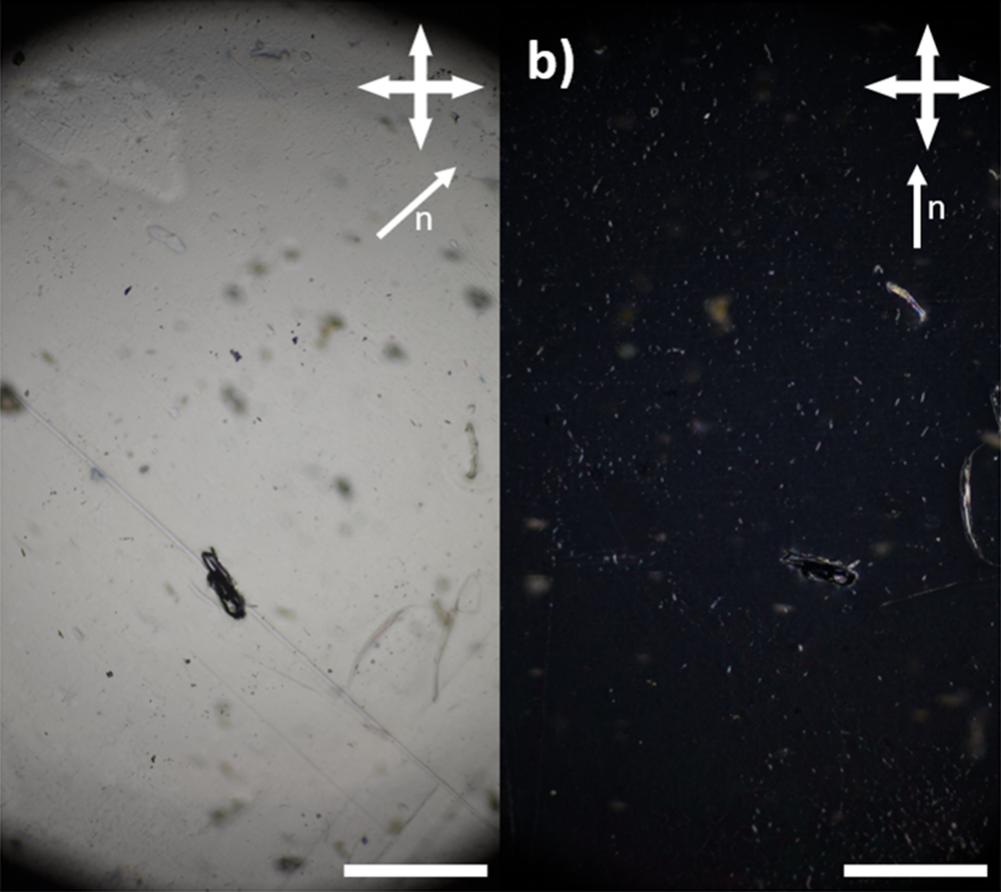
Images to show the bright and dark states (a, b, respectively) of the ∼200 μm LCEs obtained via PET-RAFT. In both cases, the scale bar represents 1 mm.

The desired alignment in the LCEs in this work is a planar alignment of the liquid crystal mesogens, with the nematic director orientated perpendicular to the longest dimension of the elastomer sample, as shown in Figure 2b. The planar alignment of the elastomer was confirmed using polarized light optical microscopy, with the polarizer and analyzer orientated at 90°. The fully cured final samples display color inversion (i.e., a bright and dark state) when rotated 45° (Figure 6), characteristic of planar alignment. This suggests that, as anticipated, the partially cured elastomer “layer” is a suitable alignment substrate for the subsequent “layers” of elastomer.

**Figure 7:**
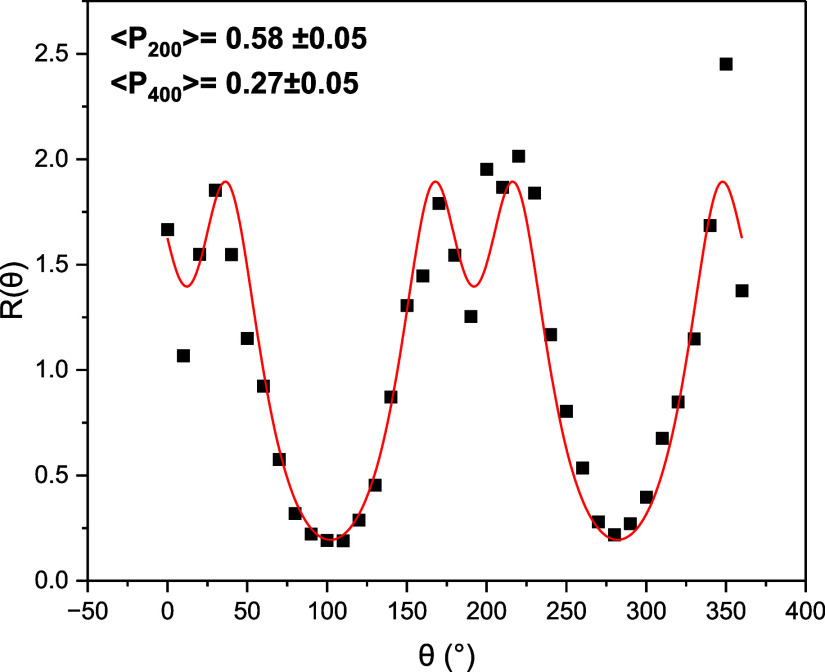
Depolarization ratio of a PET-RAFT elastomer sample of thickness ∼200 μm, determined by the 1606 cm^–1^ peak. The red line shows the fitting to the data, from which values of <*P*_200_> and <*P*_400_> were deduced.

The order parameter of the PET-RAFT elastomers was determined via Raman spectroscopy (Figure 7), in accordance with previous literature, and compared to values recorded for the same elastomers produced via free-radical polymerization.^11,33,34^ The 100 μm thick elastomers produced via free-radical polymerization exhibit order parameters in the region 0.62 ± 0.05 (Figure S9). The order parameters for the PET-RAFT LCEs were determined in two stages. First, the order parameter of the first layer of material was assessed and found to be 0.58 (±0.05), comparable to that of the free-radical LCE samples (Figure S9). The second layer of material was then added, and the cure was reinitiated. The final PET-RAFT LCE samples were then isolated, and their order parameters were measured, again yielding values of 0.58 (±0.05). This suggests a retention of the order present in the first LCE layer, and overall, a comparable order parameter for the PET-RAFT samples and free-radical LCE samples. The order parameters measured in these LCEs are comparable to those of nematic LCEs in the literature, and the nematic phase of the LCE was confirmed by X-ray scattering (Figures S11 and S12).^53−56^

### LCE Characterization

Due to the thermoset nature of these elastomers, structural analysis is limited to solid-state techniques. To assess the chemical composition of the elastomers throughout the samples, FTIR experiments were conducted on the elastomers. In these experiments, samples were taken from at least three distinct areas of each sample, ensuring that both faces of the sample were analyzed. This was done to ensure analysis both at varying positions in the sample and through the sample thickness.

**Figure 8:**
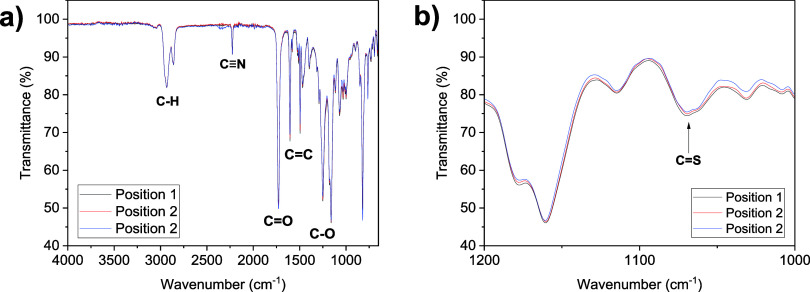
(a) Overlaid FTIR spectra taken at different positions across an ∼200 μm thick PET-RAFT elastomer sample normalized to the 2226 cm^–1^ absorbance, and (b) region of 1200–1000 cm^–1^ in the normalized FTIR spectra.

An example of the FTIR spectra obtained for distinct regions of the elastomers is displayed in Figure 8a. The spectra exhibit all the expected absorbances, notably, C–H stretching (2933 and 2860 cm^–1^), C≡N stretching (2226 cm^–1^), C=O stretching (1725 cm^–1^), C=C stretching (1605 and 1496 cm^–1^), and C–O stretching (1252 and 1163 cm^–1^). Comparisons between a PET-RAFT LCE sample and a free-radical LCE sample (Figure S13) show no notable differences.

The spectra in Figure 8 are normalized to the transmittance of the cyano group stretch at 2226 cm^–1^_,_ chosen as it is an unreactive functional group that is in a region of the spectrum where interference from other functional groups is minimal. When quantified, the strengths of the absorbances observed for the major functional groups in the spectrum are comparable (Table S1). As an example, Figure 8b shows the region of 1200–1000 cm^–1^, which highlights the absorption at 1068 cm^–1^, corresponding to the C=S bond of the RAFT agent end groups of the polymer. The transmittance values recorded for the C=S bond vary from 74.6 to 75.6%, a 1% variation that can be considered negligible. It is also of note that there is no apparent absorbance relating to unreacted acrylate groups (1636 cm^–1^) in the samples, suggesting full cure. FTIR data were also recorded at different sample depths by cutting the samples to expose bulk material (Figure S14 and Table S2), which again show no notable differences. These data suggest that the chemical composition of the PET-RAFT LCEs is comparable throughout the samples.

**Figure 9:**
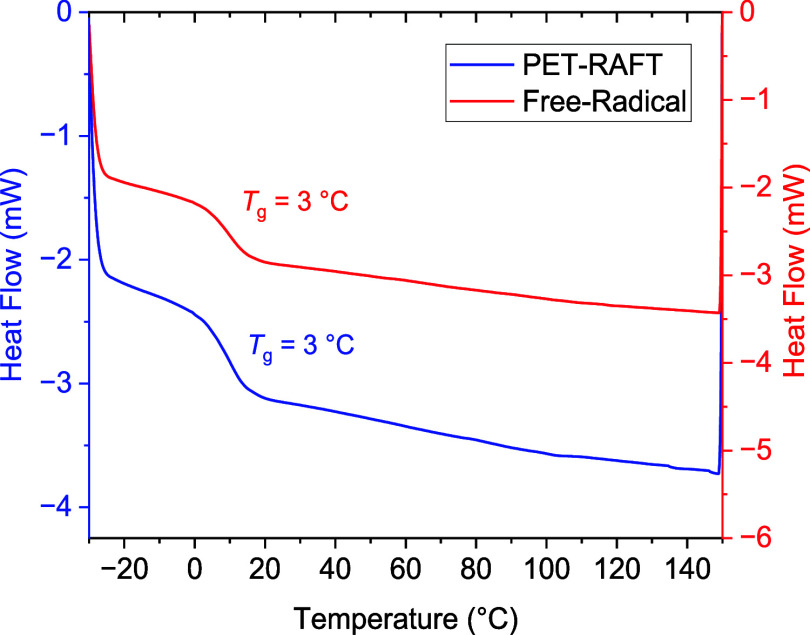
Second heating cycles of the DSC thermograms for the ∼200 μm elastomers produced through PET-RAFT (blue) and the 100 μm elastomers produced via free-radical (red) polymerization.

It is well reported that PET-RAFT (as well as RAFT polymerization in general) can lead to changes in the molecular weight distribution of polymeric products and often results in samples with a narrower dispersity.^35,36,57^ Such changes to the network have the potential to lead to changes in macroscopic material properties, such as the glass transition temperature (*T*_g_). Thus, we endeavored to examine any such effect on the properties of the LCEs. The glass transition temperature (*T*_g_) of the PET-RAFT elastomer was initially determined via DSC and compared to that of an elastomer produced via free-radical polymerization (Figure 9). Both elastomers exhibit a *T*_g_ at 3 °C (recorded as onset value on heating at 10 °C/min), suggesting that the use of PET-RAFT polymerization does not impact the *T*_g_ of the elastomers. This would indicate that the PET-RAFT elastomers could be used under comparable conditions to the elastomers synthesized via free-radical polymerization. It is of note that in both the free-radical and PET-RAFT LCEs, no further phase transitions were observed prior to thermal degradation. This lack of any other notable phase transitions is consistent with previous literature reports for the same LCEs (produced through free-radical polymerization).^10,11^

**Figure 10:**
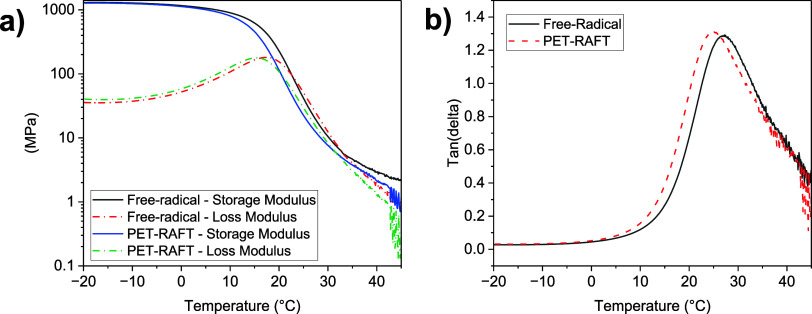
(a) Storage and loss moduli of both the PET-RAFT (∼200 μm) and free-radical (∼100 μm) LCE samples recorded on heating from −20 to 50 °C at 2 °C/min and (b) tan(δ) results for the PET-RAFT and free-radical LCE samples, recorded on heating from −20 to 50 °C at 2 °C/min.

While DSC is an appropriate means of measuring *T*_g_, it is often reported that the use of DMA can elucidate a greater quantity of information regarding the *T*_g_ of a material. It is however of note that, depending on the measurement, the nematic order of LCEs can complicate DMA analysis, as the nematic character could lead to nonlinear behavior, such as “semisoft elasticity” and in the case of these materials auxeticity.^1^ The PET-RAFT and free-radical LCE samples were subject to temperature sweep experiments at a fixed frequency and low strain (0.05%) to avoid nonlinear effects, in an attempt to glean more information with regard to any potential changes to the glass transition of the LCEs. The results of these experiments are displayed in Figure 10, and key results are detailed in Table 1.

**Table 1:** Key Results Obtained from DMA Analysis of the LCEs, as Recorded on Heating from −20 to 50 °C at 2 °C/min

sample	peak tan(δ) (°C)	peak tan(δ)	peak loss modulus (°C)	glassy modulus (MPa)
PET-RAFT	25	1.31	15	1275
free-radical	27	1.29	17	1301

The key material properties extracted from DMA affirm the comparable behavior of the PET-RAFT and free-radical LCE samples suggested by DSC data. The peak of tan(δ) and the peak of the loss modulus are regularly quoted as a means of assessing *T*_g_. In both cases, the values obtained for the PET-RAFT and free-radical LCE samples are within 2 °C of each other (25 and 27 °C for the tan(δ) peak for the PET-RAFT and free-radical LCEs, respectively, and 15 and 17 °C for the peak loss modulus). This small difference is well within tolerance for variations between the samples. In addition to these measures of *T*_g_, the overall shapes of the curves obtained from DMA are in good agreement. Notable differences in the breadth of tan(δ), in particular, could indicate more significant variations in the behavior of the LCEs; however, no such differences are observed other than the slight shift in temperature. The peak tan(δ) values and glassy modulus of the LCEs are also comparable regardless of the means of polymerization.

While the literature suggests a change in the molecular weight distribution of polymer samples may result from the use of PET-RAFT as opposed to free-radical polymerization, the evidence obtained from DSC and DMA suggests that for these systems, no such changes are observed.^35,36,57^ We hypothesize that the absence of any notable change in these results is due to the cross-linked nature of LCEs. In the literature, PET-RAFT polymerization is most commonly applied to thermoplastic polymers, in which molecular weight distributions can vary widely and be tailored by control of reaction conditions. As LCEs are cross-linked (albeit only loosely) polymer systems, the polymer chains are incorporated into a polymer network, and thus, the possibility for variation in molecular weight distribution is limited. This network almost certainly has a high molecular weight, well out of the molecular weight-dependent region of thermal properties such as *T*_g_, hence the lack of any notable differences observed.

**Figure 11:**
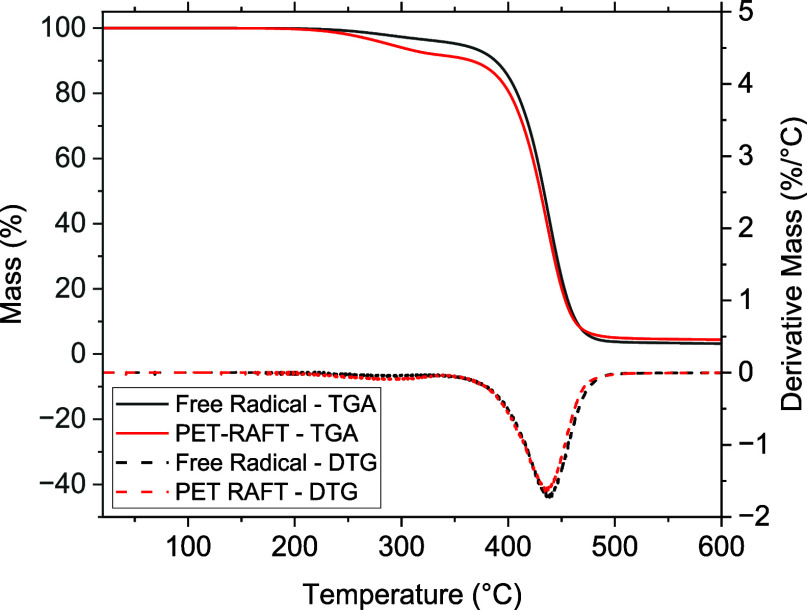
TGA thermograms (solid lines) and differential thermogravimetry (DTG) thermograms (dashed lines) for the elastomers produced via PET-RAFT (red, ∼200 μm,) and free-radical polymerization (black, ∼100 μm).

In addition to the analysis of the phase transitions of the elastomers, the thermal stability of the elastomers was evaluated by using TGA. The TGA results for a PET-RAFT elastomer and a free-radical elastomer are detailed in Table 2, with an example thermogram shown in Figure 11. The key difference in the thermal stability of the PET-RAFT and free-radical elastomers is that the PET-RAFT elastomer has a lower decomposition onset temperature than that of the free-radical elastomer (289 and 354 °C respectively). This difference is attributed to the loss of RAFT agent end groups from the polymer chain. Carbon–sulfur bonds are weaker than carbon–carbon bonds and thus would be more likely to decompose at lower temperatures than carbon–carbon bonds, as would be present at the end groups of the free-radical elastomer.^58^ The exploitation of elevated temperatures to remove RAFT end groups from polymers has also been reported in the literature.^59−62^

**Table 2:** Key Thermal Decomposition Results for the Elastomers Produced via PET-RAFT and Free-radical Polymerization^a^

sample	*T*_onset_ (°C)	*T*_inflection_ (°C)	residual mass (%)
PET-RAFT	289	437	4
free-radical	354	440	3

a *T*_onset_ denotes the temperature at which 5% of the initial sample mass is lost, and *T*_inflection_ denotes the temperature at which the maximum rate of mass loss was observed. All experiments were subject to heating from 25 to 600 °C at 10 °C/min under a nitrogen atmosphere.

The remainder of the TGA results show comparable decomposition behavior for PET-RAFT and free-radical elastomers. The major decomposition event in the region of 350–500 °C is comparable for both elastomers, as is the residual mass at 600 °C. These observations suggest that the only influence the change from free-radical polymerization to PET-RAFT polymerization has on elastomer thermal stability is the drop in the decomposition onset temperature. While this is unfavorable, there are few applications where this is likely to cause an issue.

As already mentioned, the free-radical LCE mixture used as the basis for this work is chosen because of its auxetic behavior (i.e., a negative Poisson’s ratio); this unique mechanical property offers a further test of the integrity of the double-layer sample produced via PET-RAFT.^10,11^ When subjected to strain at a given rate, this LCE is observed to initially become thinner in the *y* and *z* dimensions. However, upon reaching a threshold strain value, the LCE then begins to get thicker in one dimension (in this case, the z-dimension). This response has been characterized thoroughly in previous literature and is attributed to the emergence of biaxial order in the LCE, coinciding with an out-of-plane rotation of mesogens.^33^

**Figure 12:**
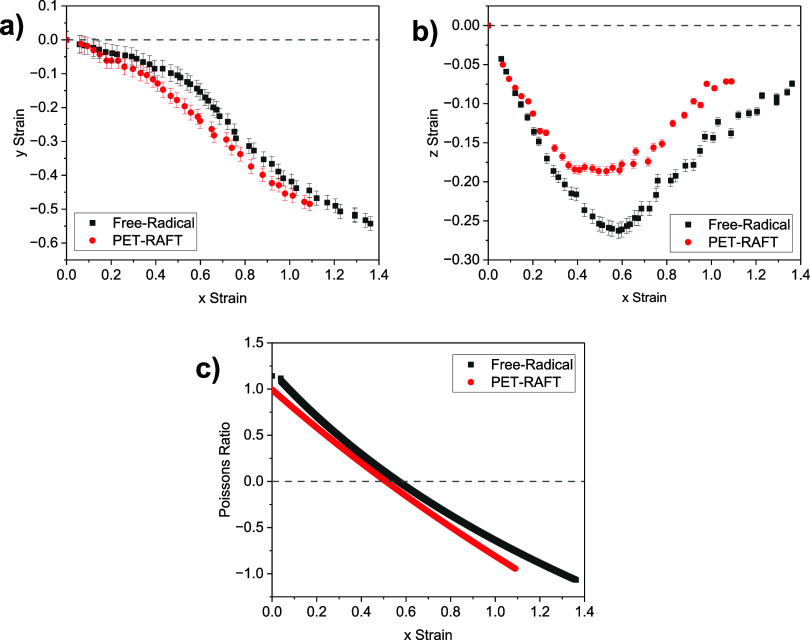
(a) *y* strain observed for the LCEs as a function of an applied *x* strain, (b) *z* strain observed for the LCE films as a function of applied *x* strain, and (c) instantaneous Poisson’s ratio calculated in the *z*-dimension for the LCE as a function of applied *x* strain. The PET-RAFT film is a double-layer of total thickness ∼200 μm, while the free radical film has a nominal thickness of 100 μm. Error bars were calculated as previously reported by Mistry et al.^10^

The presence of an auxetic response in the double-layer PET-RAFT LCEs was examined via the bespoke apparatus described in previous work within the group, and the data obtained are displayed in Figure 12. The results show that the PET-RAFT LCEs show an auxetic response in the z-dimension that is analogous to that observed for the free-radical LCEs. The threshold strain required before an auxetic response is observed (Figure 12c) was seen to be 0.51 ± 0.05 for the PET-RAFT LCEs and 0.56 ± 0.05 for the free-radical LCEs. These values are within experimental error and thus can be seen as comparable. While the strain–strain response of the LCE appears to be slightly different when examining Figure 12 a,b, for auxetic materials, the Poisson’s ratio (in this case in the z-dimension) (Figure 12c) is the crucial factor, as formally it defines the auxetic properties.^63−66^ The Poisson’s ratio data show comparable behavior between the samples, reaching a value of −0.94 for the ∼200 μm PET-RAFT LCEs and −1.06 for the ∼100 μm free-radical LCEs. It is, therefore, apparent that PET-RAFT polymerization is still suitable for producing LCEs with an auxetic response.

## Conclusions

This work has shown that PET-RAFT polymerization can be applied to the synthesis of liquid crystal elastomers, yielding nematic elastomer samples with a high-quality planar alignment. In situ FTIR studies show that the PET-RAFT mechanism can allow for the production of fully cured LCEs within 15 min of initial irradiation. This 15 min includes an induction time, in this case around 5 min, owing to the oxygen tolerance of the PET-RAFT mechanism.

We have demonstrated that the stop-start capability of PET-RAFT can be exploited to produce samples of increased thickness from that which can typically be achieved through surface alignment. This was achieved using a partially cured elastomer, wherein the reaction was stopped at 10 min, as an alignment medium, which allowed subsequent material to be aligned. Evidence gained from experiments in which the second layer of material contained no RAFT agent or photocatalyst suggests that the stop-start capability afforded by PET-RAFT results in the “layers” of LCE being chemically bonded. This could be a significant advantage over the laminate LCEs reported by Guin et al.^28,32^

The PET-RAFT elastomers displayed excellent planar alignment when examined under crossed polarizers, as confirmed by color inversion when rotated by 45°. Additionally, the PET-RAFT elastomers of 250–300 μm thickness exhibited nematic order parameters (0.58 ± 0.05) comparable to those of free-radical elastomers of 100 μm thickness (0.62 ± 0.05). The PET-RAFT and free-radical elastomers also displayed comparable glass transition temperatures according to both differential scanning calorimetry and dynamic mechanical analysis. However, the use of PET-RAFT resulted in a 65 °C decrease in the onset temperature of thermal decomposition (from 354 to 289 °C), attributed to the relative weakness of C–S bonds compared to C–C bonds. Despite this slight reduction in thermal stability, generally, the use of PET-RAFT polymerization as opposed to free-radical polymerization has a minimal impact on elastomer properties.

The application of PET-RAFT polymerization to LCEs could create several possibilities for future research. As demonstrated in this work, the increased thickness possible via PET-RAFT in principle allows for the production of films of elastomers that can achieve the forces required for LCEs to fulfill their potential for proposed applications. Additionally, we have shown that the stop-start capability of PET-RAFT can be exploited in LCEs, and thus future investigations could investigate what else this stop-start ability can allow us to achieve. Examples may include complex patterns consisting of isotropic and liquid crystal phases in one chemically bound elastomer. PET-RAFT polymerization therefore presents an interesting opportunity for future LCE research.

## Data Availability

The data underlying this study are openly available in the data set associated with “Toward Monodomain Nematic Liquid Crystal Elastomers of Arbitrary Thickness Through PET-RAFT Polymerization”, available at 10.5518/1393.

## Supporting Information Available

Additional experimental details and synthetic procedure; ^1^H and ^13^C NMR spectra for the synthesized materials; DSC thermograms and polarized optical microscopy images to confirm the phase transitions of the synthesized materials; Raman spectroscopy depolarization ratio fitting for LCEs; additional FTIR spectral data acquired from the curing studies undertaken; SAXS and WAXS data for PET-RAFT and free-radical LCE samples; overlaid FTIR spectra comparing the free-radical and PET-RAFT LCE samples, as well as overlaid FTIR spectra throughout the PET-RAFT sample thickness; transmittance values for significant peaks in the FTIR spectra of PET-RAFT samples, to show the comparable composition of the sample at numerous positions; and sample thickness measurements for PET-RAFT and free-radical LCEs (PDF)

## Abbreviations

6OCB: 4′-Hexyloxy-(1,1′-biphenyl)-4-carbonitrile
6OCB–OH: 4′-(6-hydroxyhexyloxy)-[1,1′-biphenyl]-4-carbonitrile
A6OCB: 6-(4-Cyano-biphenyl-4′-yloxy)hexyl acrylate
DCM: dichloromethane
DDMAT: 2-(((Dodecylthio)carbonothioyl)thio)-2-methylpropanoic acid
DMA: Dynamic Mechanical Analysis
DMF: dimethylformamide
DSC: differential scanning calorimetry
EHA: 2-ethylhexyl acrylate
FTIR: Fourier-transform infrared spectroscopy
Ir(PPy)3: Tris(2-phenylpyridine)iridium
LCE: liquid crystal elastomer
MBF: methyl benzoylformate
NMR: nuclear magnetic resonance
PET-RAFT: photoinduced electron/energy transfer reversible addition–fragmentation chain transfer
RAFT: reversible addition–fragmentation chain transfer
RDRP: reversible deactivation radical polymerization
RM82: 2-Methyl-1,4-phenylene bis(4-((6-(acryloyloxy)hexyloxy)benzoate
*T*_g_: glass transition temperature
TGA: thermogravimetric analysis
THF: tetrahydrofuran
*T*_inflection_: temperature of maximum rate of thermal decomposition
*T*_onset_: temperature at which 5% of original mass is lost.
