# Supplementary material for: Toward Monodomain Nematic Liquid Crystal Elastomers of Arbitrary Thickness through PET-RAFT Polymerization
Source: Macromolecules. 2024 May 28;57(11):5218–29. doi: 10.1021/acs.macromol.4c00245 (PMC11171763; doi:10.1021/acs.macromol.4c00245)
Supplement: Supplementary file 1 — ma4c00245_si_001.pdf [file ma4c00245_si_001.pdf]

# Toward Monodomain Nematic Liquid Crystal Elastomers of Arbitrary Thickness Through PET-RAFT Polymerization

Stuart R. Berrow[1*], Richard J. Mandle[1,2], Thomas Raistrick[1], Matthew Reynolds[1] and Helen F. Gleeson[1]

[1]School of Physics and Astronomy, University Of Leeds, LS2 9JT

[2]School of Chemistry, University of Leeds, LS2 9JT

## Electronic Supplementary Information

**Supporting Information available**: General experimental information, synthetic Procedures for synthesis of 6-(4-Cyano-biphenyl-4′-yloxy)hexyl acrylate, full experimental procedure for elastomer mould fabrication, structural and thermal characterisation of 4’-(6-hydroxyhexyloxy)-[1,1’-biphenyl]-4-carbonitrile and 6-(4-Cyano-biphenyl-4′-yloxy)hexyl acrylate, Comparison of FTIR spectra for free-radical and PET-RAFT LCEs, FTIR conversion studies spectra, Raman depolarization data for free-radical LCE and the first layer of PET-RAFT LCE, X-ray scattering data, LCE thickness measurements.

# Table of Contents

General Experimental Information .......... 2
Preparation of 6-(4-Cyano-biphenyl-4′-yloxy)hexyl acrylate (A6OCB) .......... 4
Elastomer Cell Fabrication .......... 5
Structural Analysis of 4’-(6-hydroxyhexyloxy)-[1,1’-biphenyl]-4-carbonitrile .......... 6
Structural Analysis of 6-(4-Cyano-biphenyl-4′-yloxy)hexyl acrylate .......... 8
Monomer/Precursor Characterisation .......... 10
Raman Order Parameters .......... 12
Additional Evidence for FTIR Cure Studies .......... 13
X-ray Scattering .......... 14
FTIR Analysis .......... 16
LCE Thickness Measurements .......... 17
References .......... 18

## General Experimental Information

### Structural Analysis

Nuclear magnetic resonance spectra were recorded using a Bruker AVANCE III (400 MHz) NMR spectrometer (Bruker UK Ltd., Coventry, UK) at 298 K and referenced to TMS. NMR spectra were viewed and analysed using Bruker Topspin NMR software.

Mass Spectrometry was recorded using a Bruker Amazon Speed (Bruker UK Ltd., Coventry, UK) mass spectrometer, fronted by a Dionex UltiMate 3000 uHPLC system (Thermo Scientific, Loughborough, UK). Samples submitted as solutions in acetonitrile as 1mg/mL concentration. Data was collected in positive mode, and samples were ionised using electrospray ionisation. The accuracy of the instrument is ±1 $gmol^{-1}$.

### Optical Microscopy Measurements

Polarised light optical microscopy was performed using a Leica DM2700P polarised light microscope (Leica Microsystems (UK) Ltd., Milton Keynes, UK), equipped with 5x and 50x magnification objectives. For elastomer samples, these investigations were used to assess the quality of planar alignment of the samples. Elastomers were mounted on a glass slide and analysed under ambient conditions.

For phase identification of the liquid crystalline materials, a Mettler Toledo FP82HT Hot Stage (Mettler-Toledo Ltd., Leicester, UK), controlled by a Mettler Toledo FP90 central processor (Mettler-Toledo Ltd., Leicester, UK) was used to control the temperature of the sample. In this case, the sample was mounted between a glass microscope slide and a glass cover slip. Images were recorded using a Nikon D3500 Digital Camera (Nikon UK Ltd., Surbiton, UK), using DigiCamControl software.

### Order Parameter Measurements

The nematic order parameter of the liquid crystal elastomers was recorded as described previously.[1,2] In brief, the full depolarisation ratio was recorded in 10˚ steps, allowing the order parameters to be deduced from fits to the depolarisation ratio data. The 1606 $cm^{-1}$ Raman mode was selected which is associated with the C-C stretch of the biphenyl rings of the mesogenic units. The order parameter was deduced with an accuracy of ±0.05. Raman spectra were recorded using a Renishaw inVia Raman spectrometer, equipped with a 532 nm, 50 mW solid state laser, and a Leica DM2700P polarised light microscope equipped with a rotating stage. Measurements were using a 20x objective with 1% laser power.

### Thermal Analysis

Differential scanning calorimetry (DSC) measurements were performed using a TA Instruments Q20 DSC instrument (TA Instruments, Wilmslow UK), equipped with a RCS90 Refrigerated cooling system (TA Instruments, Wilmslow UK). The instrument was calibrated against an Indium standard, and data was processed using TA Instruments Universal Analysis Software. Samples were analysed under a nitrogen atmosphere, in hermetically sealed aluminium TZero crucibles (TA Instruments, Wilmslow, UK) and subjected to 2 analysis cycles. For the analysis of the synthesised monomer and intermediate, each cycle consisted of: a heating phase from 0–140 °C at a heating rate of 10 °C/min, an isothermal phase at 140 °C for 2 minutes, a cooling phase from 140–0 °C at 10 °C/min, and an isothermal phase at 0 °C for 2 minutes. These experiments sought to determine phase transitions temperatures of the materials, and all temperature values are reported as onset values. For elastomer analysis, each cycle consisted of: a heating phase from -50–150 °C at a heating rate of 10 °C/min, and isothermal phase at 150 °C for 2 minutes, a cooling phase

from 150 – -50 °C at 10 °C/min, and an isothermal phase at -50 °C for 2 minutes. The glass transition temperatures of the polymers were recorded as the onset value, on the heating phase of the second cycle.

Thermogravimetric analysis (TGA) was performed using a TA Instruments TGA Q50 (TA Instruments, Wilmslow UK), and the data processed using TA Instruments Universal Analysis Software. Samples were analysed in platinum crucibles under a nitrogen atmosphere, and were subject to heating from 25–600 °C at 10 °C/min. The decomposition onset temperature ($T_{onset}$) was reported as the temperature at which 5% of the original sample mass had been lost. The inflection temperature ($T_{inflection}$) was reported as the temperature at which peak mass loss rate was achieved according to the first derivative off mass with respect to temperature.

Dynamic Mechanical Analysis (DMA) was performed using a TA Instruments DMA 850 (TA Instruments, Wilmslow UK), equipped with a liquid nitrogen cooling system, and the data processed using TRIOS software. Samples of dimensions approximately 10 mm x 2 mm x 100 µm were subject to temperature sweeps using film clamps. In a typical measurement, samples were analysed over the range of -20-50 °C, with a heating/cooling rate of 2 °C/min. A strain of 0.05% was applied at a frequency of 1 Hz.

**Fourier-Transform Infra-red Spectroscopy (FTIR)**

FTIR analysis on the samples was conducted using a Perkin Elmer FT-IR Spectrum One model in conjunction with ATR diamond cell (Beaconsfield, UK). Spectra were recorded between 4000-650 $cm^{-1}$ collecting 100 scans for each experiment.

In a typical FTIR curing investigation, a sample of the uncured LCE mixture was applied to the FTIR spectrometer crystal and covered with a Melinex® ST725 substrate. This substrate had been treated with poly(vinyl alcohol), to induce planar alignment, and had been pre-adhered to two Melinex® 401 spacers of 100 µm thickness, in order to best mimic the conditions used for polymerisation (see **Elastomer Cell Fabrication**). It is of note that all spectra were recorded at room temperature, well within the nematic phase of the precursor mixture, and again mimicking the conditions applied during polymerisation. An FTIR spectrum of the precursor mixture was recorded prior to irradiation. The samples were then subjected to irradiation at 350 nm (2.5 $Wcm^{-2}$), and spectra recorded at 5-minute intervals for 60 minutes.

**X-Ray Scattering**

2D Small angle (SAXS) and wide angle (WAXS) X-ray scattering experiments were performed on an Anton Paar SAXSpoint 5.0 system (K-α Cu source, λ=1.5418 Å) with a Dectris EIGER2 R 1M (1028 pixel x 1062 pixel array). Measurements were performed at room temperature averaging 5 frames with exposures times of 60 s. A 2 mm beam size was used and the measurements were run using a beam stop-less set-up. A background scan was performed for both the SAXS and WAXS detector position which was subtracted from the measurements to minimise contributions from intrinsic background scattering and the Mylar protective film in front of the detector. 2D data reduction was performed by radially integrating the 2D patterns whilst masking the central contribution related to the non-scattered X-ray beam.

## Mechanical Analysis

Mechanical measurements were conducted using bespoke equipment designed and manufactured in-house, full specifications for which can be found in previous work.[2,3] This apparatus consists of two actuators and a load cell, enclosed within a temperature-controlled environment, and is equipped with optics that enable images of the sample to be recorded both via optical microscopy and polarising optical microscopy simultaneously. In this work, samples of 20 mm x 2 mm were analysed at room temperature. The initial gap between the actuators was 16.5 mm, and the samples were subject to strain steps of 0.5 mm at 10 minute intervals, until the sample failed. The samples were strained perpendicular to the initial nematic director, as displayed in **Figure S1**.

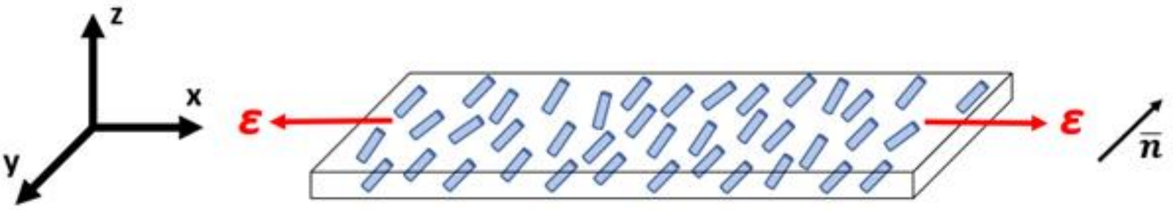


***Figure S1*** *- A diagram to describe the procedure for the mechanical testing used to monitor the auxetic behaviour of the LCEs, showing strain (ε) being applied perpendicular to the initial nematic director ($\bar{n}$). The mesogenic units (both side-chain and cross-linker are shown schematically as blue cylinders with their average direction (the director) indicated. The LCE backbone is not shown.*

## Preparation of 6-(4-Cyano-biphenyl-4′-yloxy)hexyl acrylate (A6OCB)

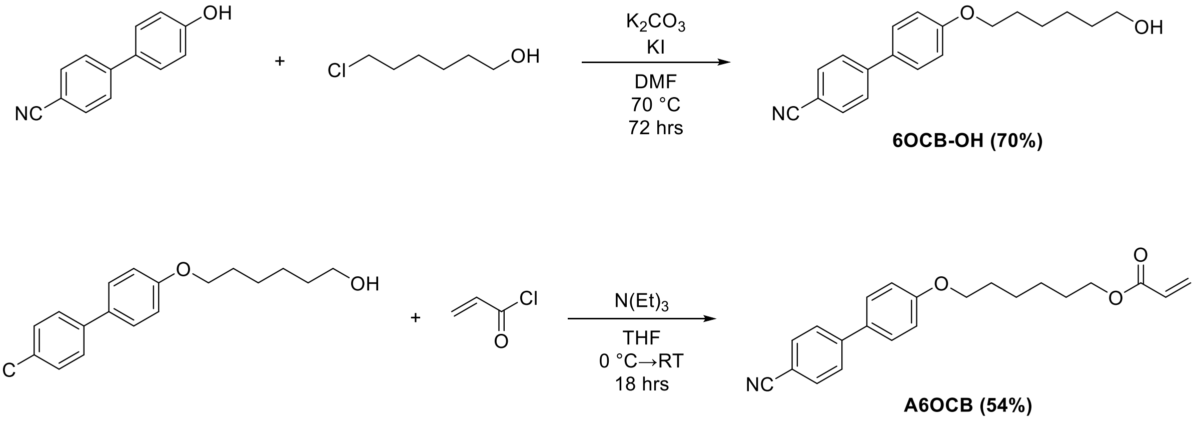


***Figure S2*** *- The synthetic pathway used to synthesise A6OCB.*

### 4’-(6-hydroxyhexyloxy)-[1,1’-biphenyl]-4-carbonitrile (6OCB-OH)

The synthesis of 4’-(6-hydroxyhexyloxy)-[1,1’-biphenyl]-4-carbonitrile was adapted from Hayata *et al.*[4] To a solution of 4-Cyano-4'-hydroxybiphenyl (6.61 g, 33.9 mmol) in DMF (50 mL) was added potassium carbonate (7.03 g, 50.9 mmol) and potassium iodide (0.23 g, 1.4 mmol), and the resulting suspension stirred at room temperature for 1 hour. To this suspension was added dropwise a solution of 6-chloro-1-hexanol (6.02 g, 44.1 mmol) in DMF (20 mL), and the resulting mixture stirred at 70 °C for 72 hours. The reaction mixture was then filtered under gravity, and the filtrate diluted in chloroform (2x 100 mL). The resulting solution was washed with water (100 mL), 1M sodium hydrogen carbonate solution

(2x 100 mL) and saturated brine solution (100 mL) and the organic layer dried over sodium sulphate. The solvent was then removed under reduced pressure, and the resulting colourless solid recrystallised from 1:1 n-hexane:ethyl acetate yielding 4'-(6-hydroxyhexyloxy)-[1,1'-biphenyl]-4-carbonitrile (**6OCB-OH**) as colourless crystals (7.05 g, 70 %). $^{1}$H NMR (400 MHz, $CDCl_3$) $\delta_H$(ppm): 1.39-1.51 (m, 4H, $-CH_2-$), 1.51-1.59 (m, 2H, $-CH_2-$), 1.72-1.81 (m, 2H, $-CH_2-$), 3.60 (t, 2H, *J*= 7Hz, $-CH_2$-O-), 3.94 (t, 2H, J= 7Hz, $-CH_2$-O-), 6.92 (dt, 2H, *J*= 9,3 Hz, Ar-H), 7.45 (dt, 2H, *J*= 9,3 Hz, Ar-H), 7.55-7.63 (m, 4H, Ar-H). $^{13}$C {$^{1}$H} NMR (400 MHz, $CDCl_3$) $\delta_C$(ppm): 25.6 ($-CH_2-$), 25.9 ($-CH_2-$), 29.2 ($-CH_2-$), 32.4 ($-CH_2-$), 62.3 (-O-$CH_2$-), 68.0 (-O-$CH_2$-), 110.1 (Ar C-R), 115.1 (Ar C-H), 119.2 (-C≡N), 127.2 (Ar C-H), 128.4 (Ar C-H), 131.4 (Ar C-R), 132.6 (Ar C-H), 145.3 (Ar C-R), 159.7 (Ar C-OR). LCMS m/z: 295.51 ($M^+$) (Purity 99.9+% (only one peak detected)). Phase transitions: 94 °C (Melting Point), 109 °C (clearing point).

### 6-(4-Cyano-biphenyl-4′-yloxy)hexyl acrylate (A6OCB)

The synthesis of 6-(4-Cyano-biphenyl-4′-yloxy)hexyl acrylate was adapted from Hayata et al.[4] A solution of 4'-(6-hydroxyhexyloxy)-[1,1'-biphenyl]-4-carbonitrile (5.00 g, 16.9 mmol) and triethylamine (2.06 g, 20.3 mmol) in THF (50 mL) was cooled to 0 °C. To this solution was added dropwise a solution of acryloyl chloride (1.84 g, 20.3 mmol) in THF (10 mL). The resulting mixture as stirred at 0 °C for 1 hour, before stirring overnight at room temperature. The solvent was removed under reduced pressure, and the resulting solid dissolved in chloroform (100 mL), before washing with water (100 mL), 1M sodium hydrogen carbonate solution (100 mL) and saturated brine solution (100 mL). The organic layer was dried over sodium sulphate, and the solvent removed under reduced pressure. The resulting solid was recrystallised from 1:1 n-hexane:ethyl acetate to obtain 6-(4-Cyano-biphenyl-4′-yloxy)hexyl acrylate (**A6OCB**) as a colourless solid (3.28 g, 54 %). $^{1}$H NMR (400 MHz, $CDCl_3$) $\delta_H$(ppm): 1.35-1.52 (m, 4H, $-CH_2-$), 1.61-1.70 (m, 2H, $-CH_2-$), 1.72-1.90 (m, 2H, $-CH_2-$), 3.94 (t, 2H, *J*= 7Hz, $-CH_2$-O-), 4.10 (t, 2H, *J*= 7Hz, $-CH_2$-O-), 5.74 (dd, 1H, *J*= 10,1 Hz, $=CH_2$), 6.05 (dd, 1H J= 15,10 Hz, -CH=), 6.33 (dd, 1H, *J*= 10,1 Hz, $=CH_2$), 6.92 (dt, 2H, *J*= 9,3 Hz, Ar-H), 7.45 (dt, 2H, *J*= 9,3 Hz, Ar-H), 7.54-7.64 (m, 4H, Ar-H). $^{13}$C {$^{1}$H} NMR (400 MHz, $CDCl_3$) $\delta_C$(ppm): 25.7 ($-CH_2-$), 28.6 ($-CH_2-$), 29.1 ($-CH_2-$), 64.5 (-O-$CH_2$-), 68.0 (-$CH_2$-O), 110.1 (Ar C-R), 115.1 (Ar C-H), 119.2 (-C=N), 127.1 (Ar C-H), 128.3 (Ar C-H), 128.6 (C=C), 130.6 (C=C), 131.4 (Ar C-R), 132.6 (Ar C-H), 145.3 (Ar C-R), 159.8 (Ar C-OR), 166.4 (C=O). LCMS m/z: 349.64 ($M^+$), 277.50 ($[M-C_3H_3O_2]^+$) (Purity 99.9+% (only one peak detected)). Phase Transitions: 66 °C (Melting point).

## Elastomer Cell Fabrication

A glass microscope slide (7.5 cm x 2.5 cm x 1 mm), and a Melinex® ST725 substrate (7 cm x 2.5 cm x 250 µm) (DuPont Teijin Films, Redcar, UK) were spin coated on one surface with an aqueous 0.5 wt % polyvinyl alcohol (PVA) solution, which was uniaxially rubbed when dry. These two substrates were then assembled into a mould, including Melinex® 401 spacers (7.5 cm x 0.2 cm x 100 µm) (DuPont Teijin Films, Redcar, UK) and UVS-91 adhesive, so that the PVA rubbed surfaces were the inner surfaces of the constructed cell. The adhesive was then cured by irradiation at 350 nm (2.5 Wcm-2) for 10 minutes, to yield the constructed elastomer cell.

## Structural Analysis of 4’-(6-hydroxyhexyloxy)-[1,1’-biphenyl]-4-carbonitrile

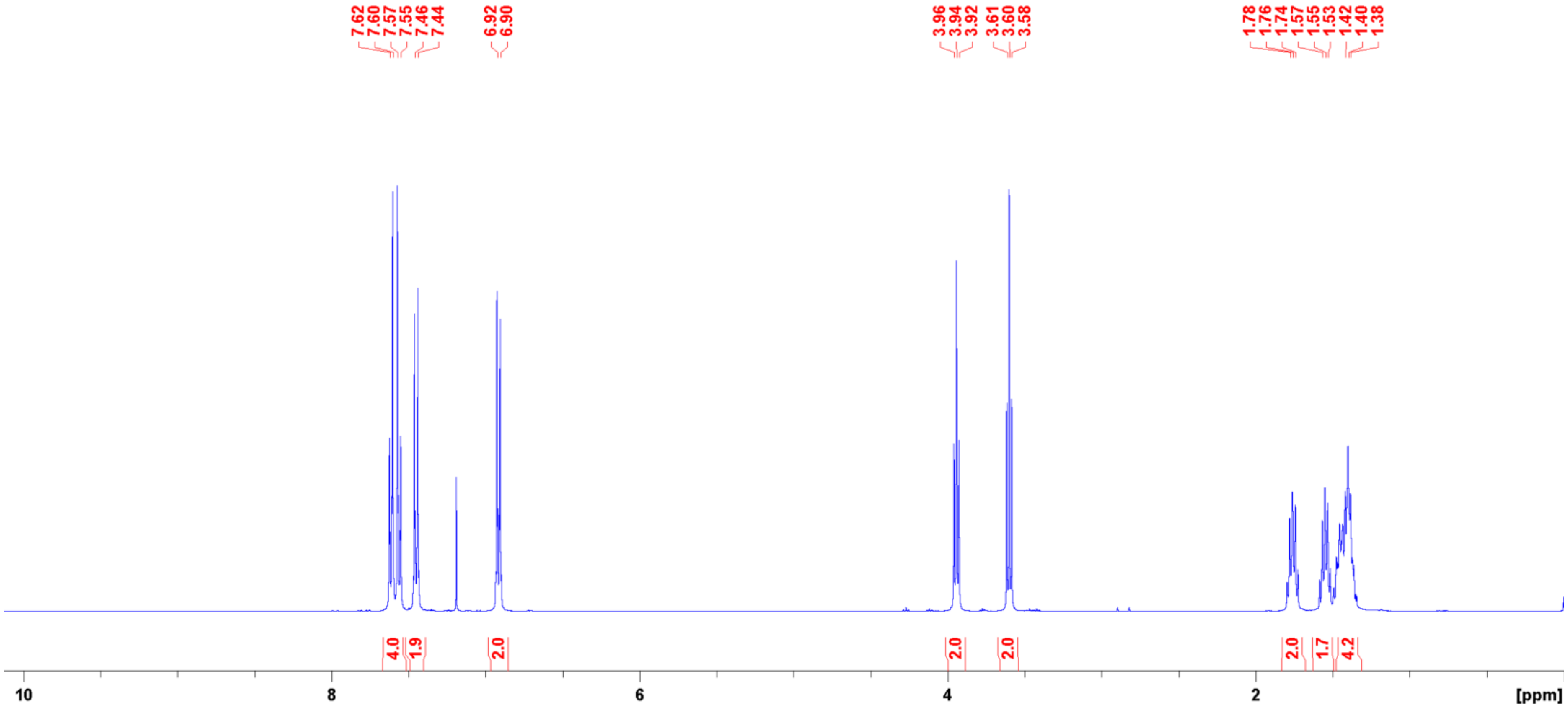


***Figure S3*** *– $^1$H NMR spectrum of 4’-(6-hydroxyhexyloxy)-[1,1’-biphenyl]-4-carbonitrile, recorded in $CDCl_3$.*

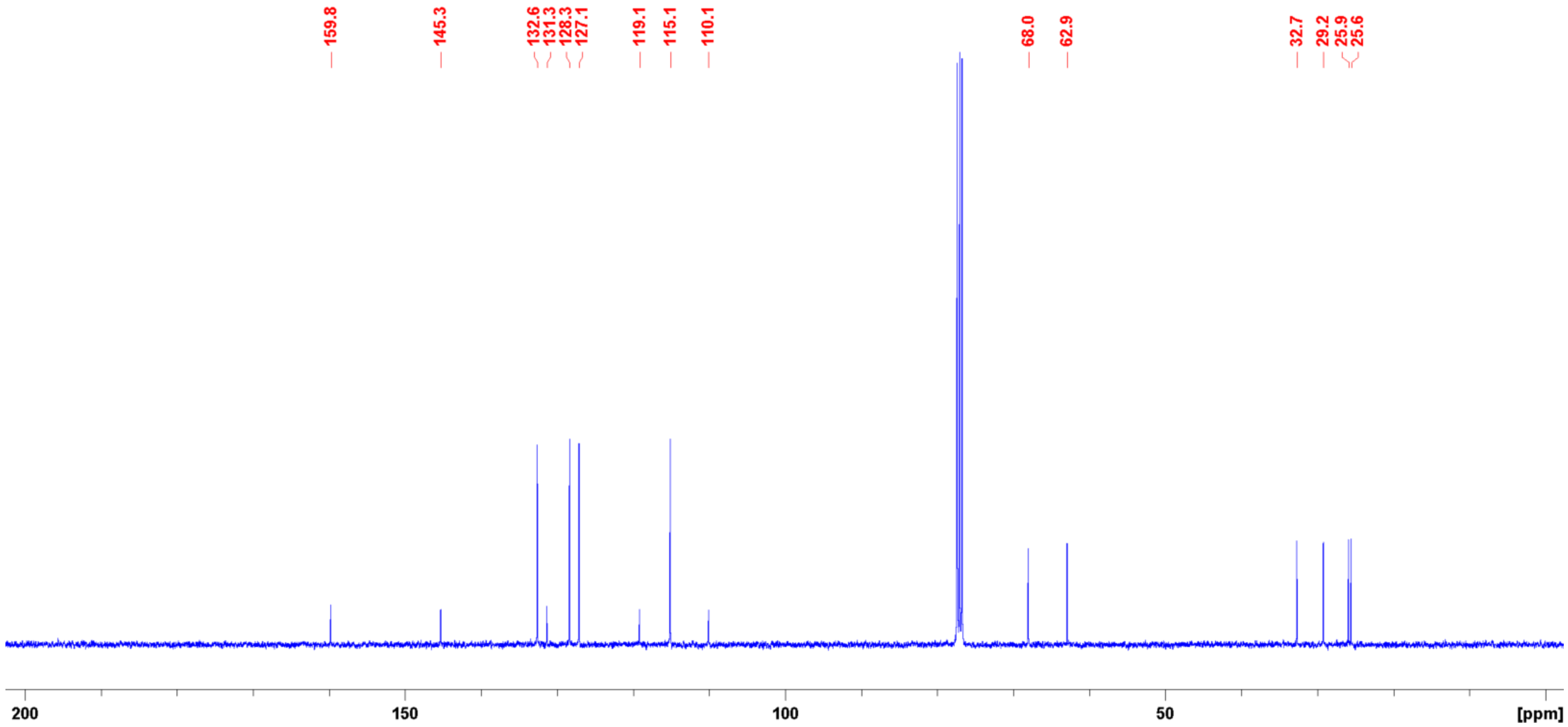


***Figure S4*** *– $^{13}$C NMR spectrum of 4'-(6-hydroxyhexyloxy)-[1,1'-biphenyl]-4-carbonitrile, recorded in* $CDCl_3$.

## Structural Analysis of 6-(4-Cyano-biphenyl-4′-yloxy)hexyl acrylate

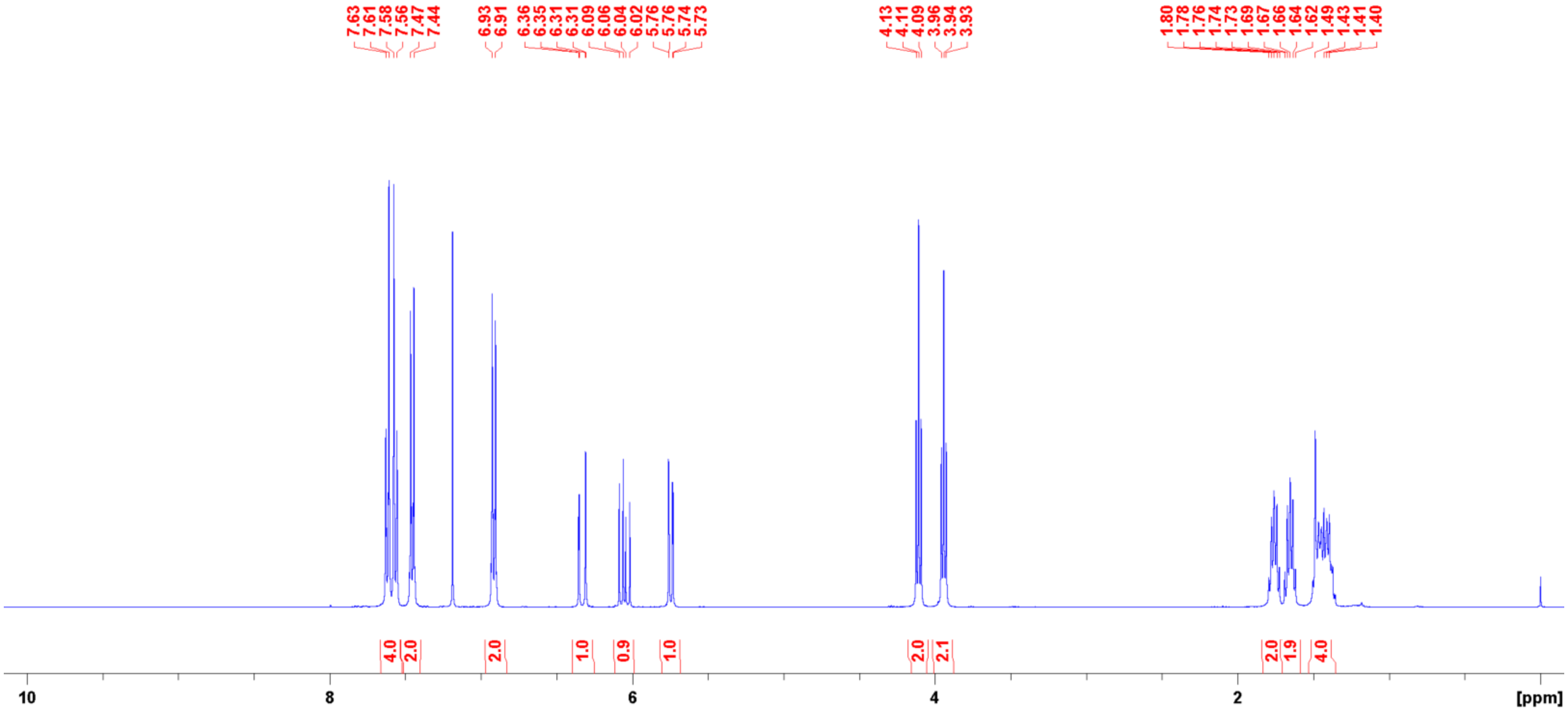


***Figure S5*** *– $^{1}$H NMR spectrum of 6-(4-Cyano-biphenyl-4'-yloxy)hexyl acrylate, collected in $CDCl_3$.*

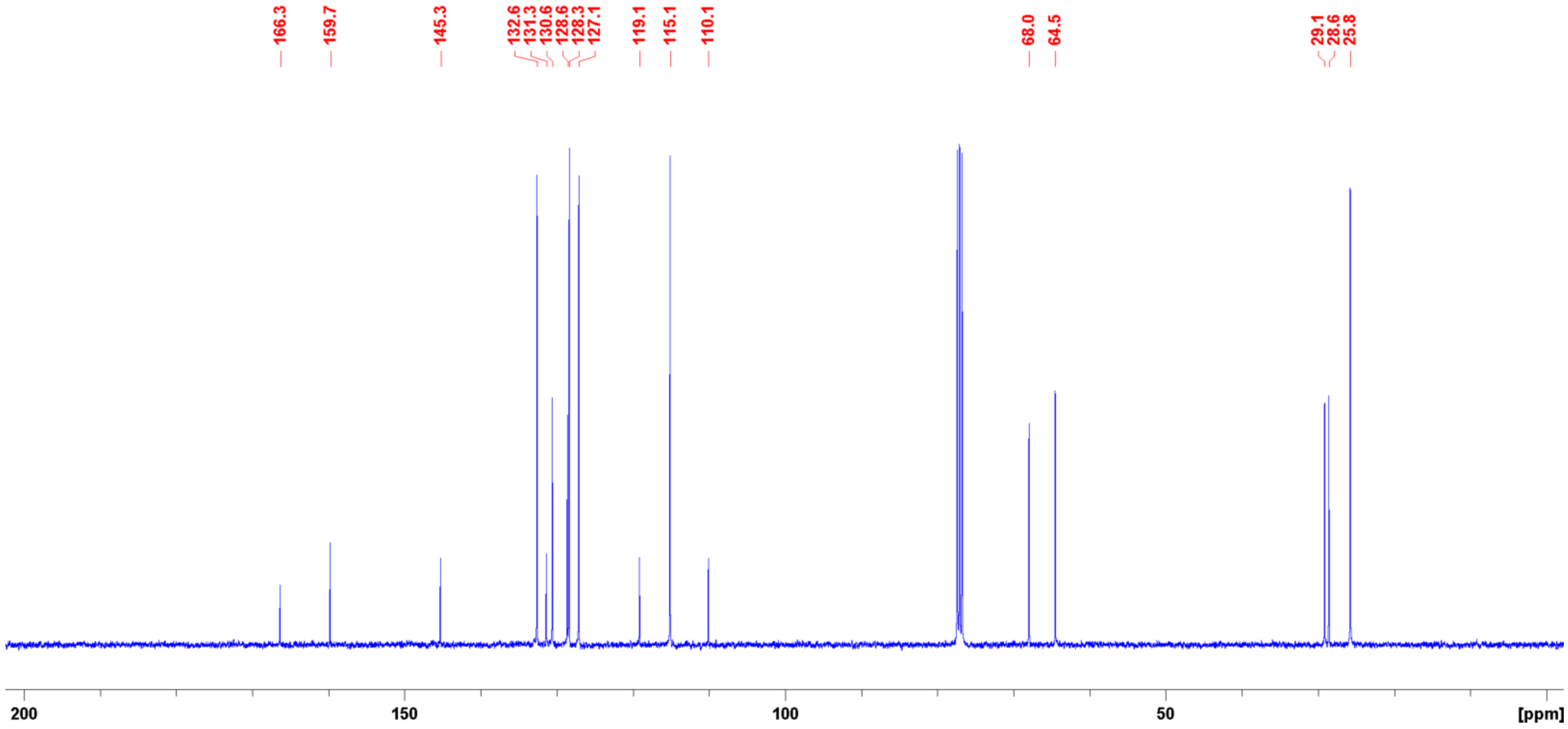


***Figure S6*** – *$^{13}C$ NMR spectrum of 6-(4-Cyano-biphenyl-4'-yloxy)hexyl acrylate, collected in $CDCl_3$.*

## Monomer/Precursor Characterisation

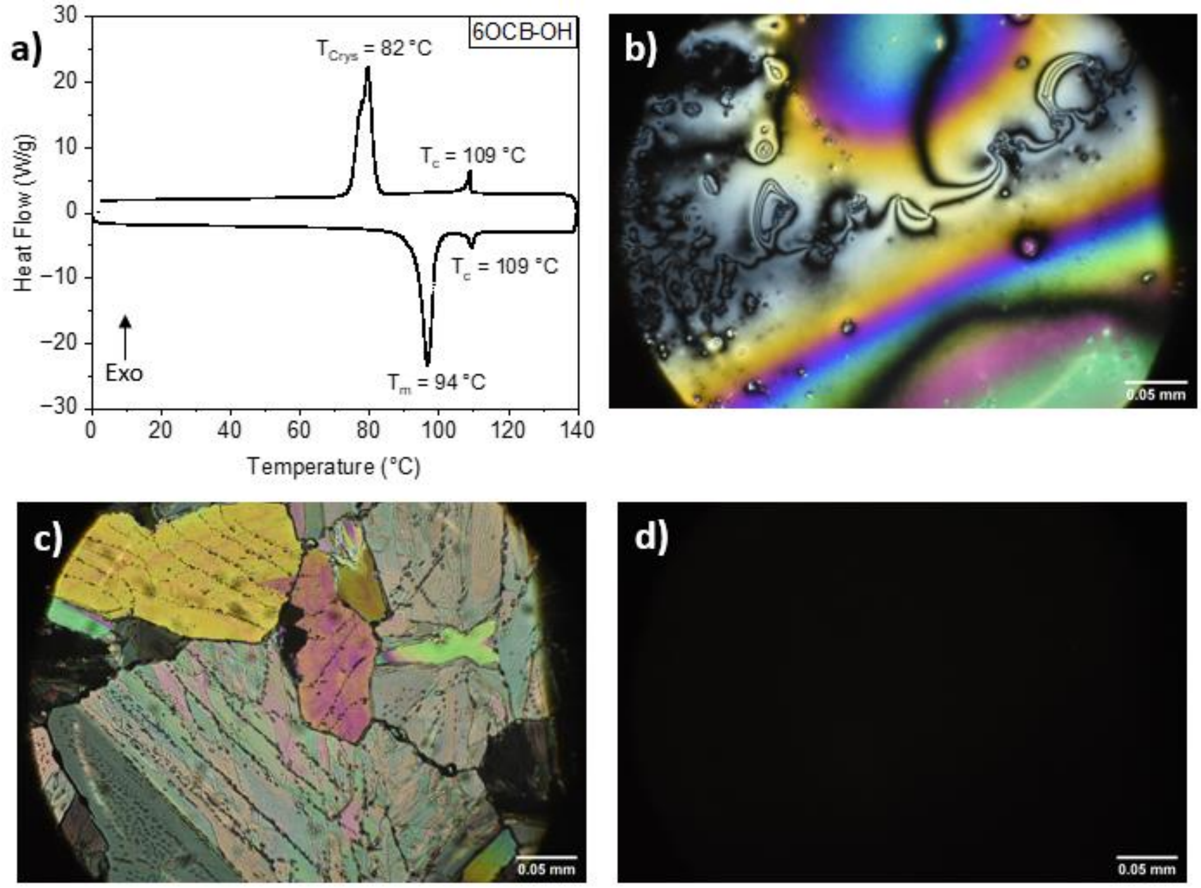


***Figure S7*** *- a) An example DSC thermogram for the synthesised 6OCB-OH, b) the Schlieren texture observed for the nematic phase of 6OCB-OH at 108 °C, c) the crystal phase observed at 80 °C, and d) the isotropic phase observed at 120 °C. In all cases, the scale bar represents 0.05 mm, and all images were recorded on cooling. ($T_{Cryst}$ denotes the crystallisation temperature, $T_c$ denotes clearing temperature, and $T_m$ denotes melting temperature)*

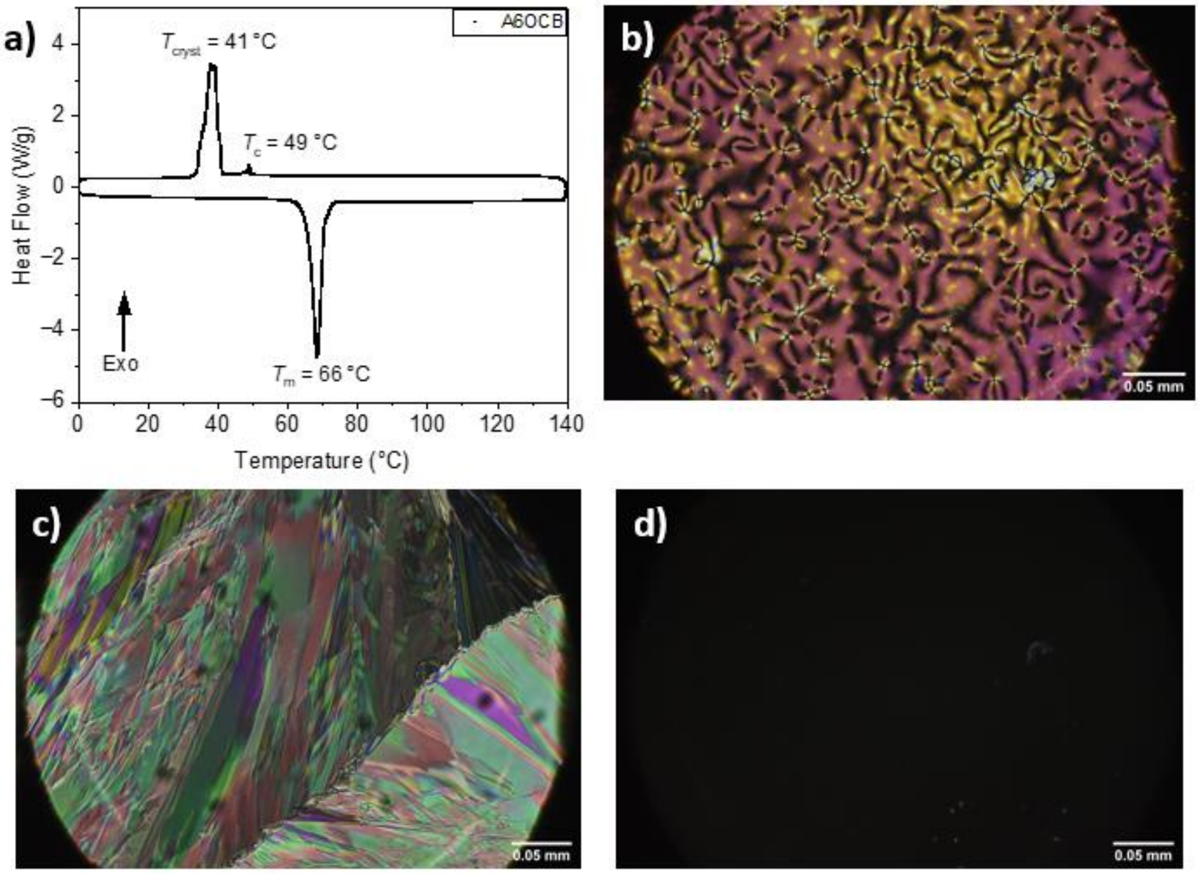


***Figure S8** – a) An example DSC thermogram for the synthesised A6OCB, b) the Schlieren texture observed for the nematic phase of A6OCB at 47 °C, c) the crystal phase observed at 25 °C, and d) the isotropic phase observed at 100 °C. In all cases, the scale bar represents 0.05 mm, and all images were recorded on cooling. ($T_{Cryst}$ denotes the crystallisation temperature, $T_c$ denotes clearing temperature, and $T_m$ denotes melting temperature)*

**Raman Order Parameters**

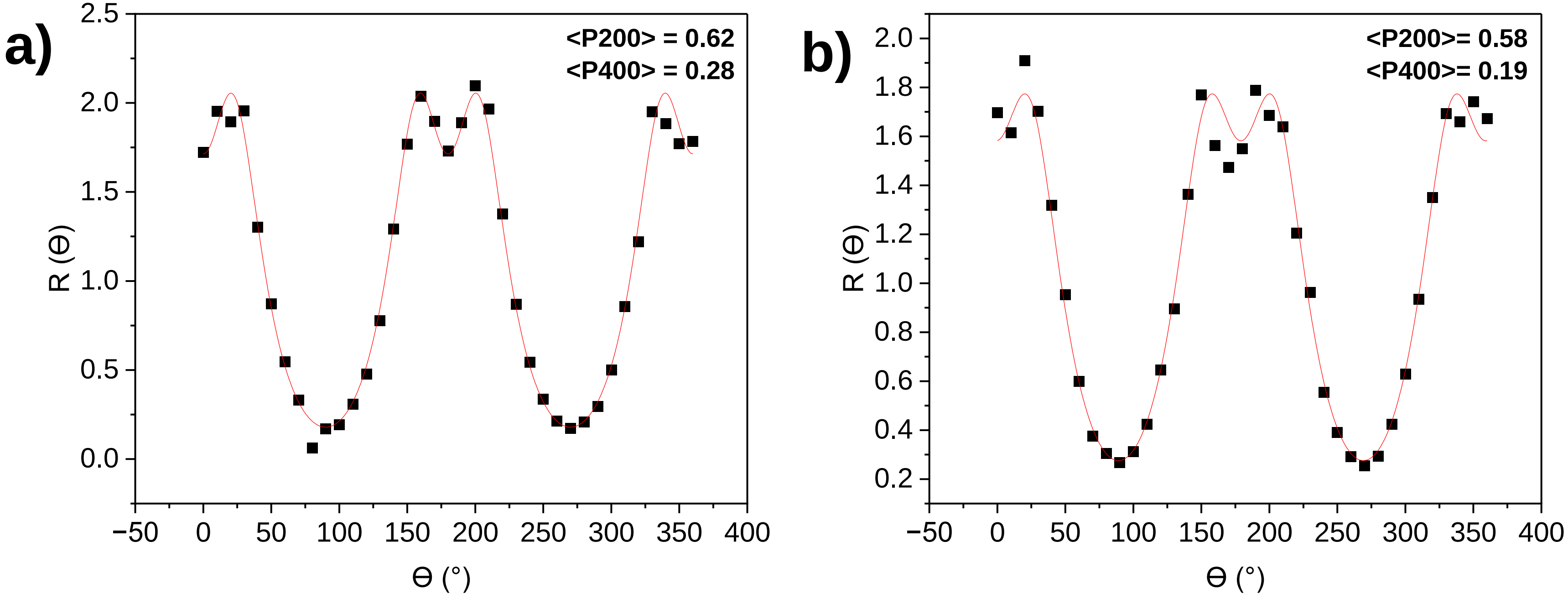


***Figure S9*** *– The depolarization ratio of a) a free-radical LCE sample (100 µm thickness) and b) the initial layer of a PET-RAFT LCE sample (approximately 100 µm thickness). In both cases, the depolarization ratios were determined by the 1606 cm$^{-1}$ peak in the Raman spectrum, and the red curve shows the fitting data from which* <$P_{200}$> *and* <$P_{400}$> *were recorded.*

## Additional Evidence for FTIR Cure Studies

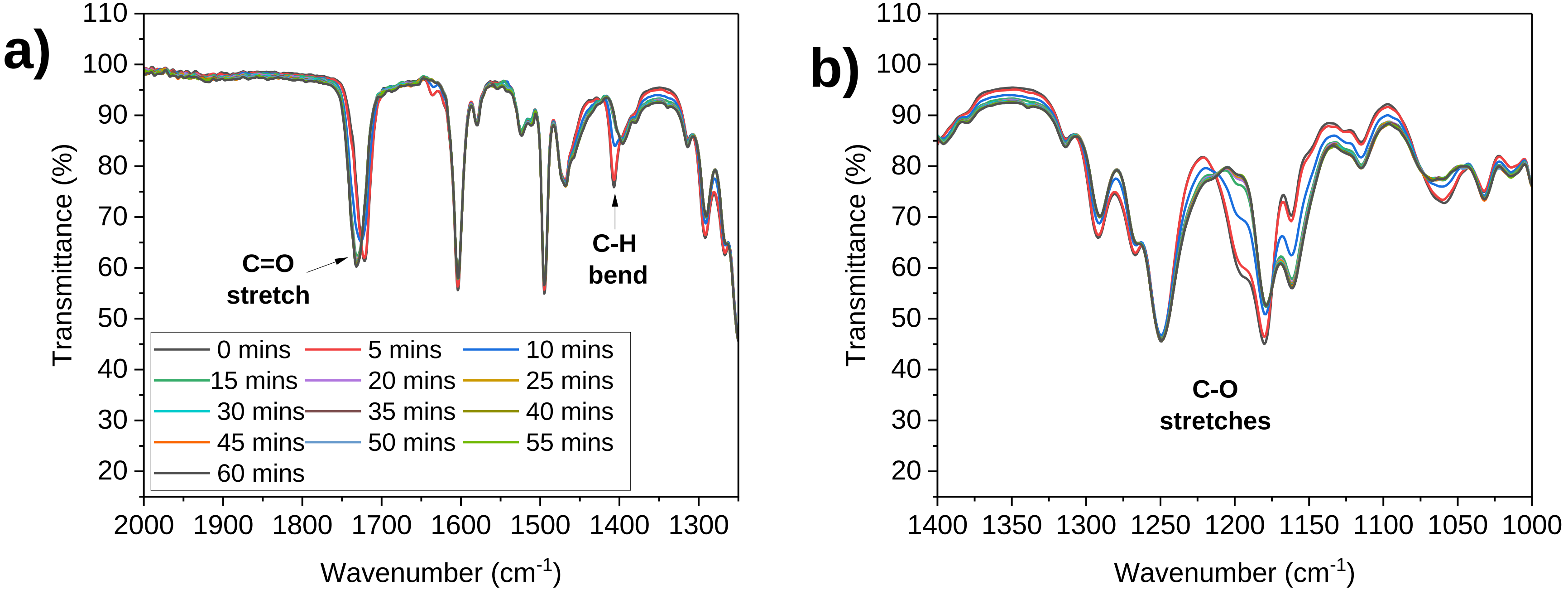


***Figure S10*** *– FTIR spectra showing the regions of a) 2000-1250 cm$^{-1}$ and b) 1000-1400 cm$^{-1}$ for the PET-RAFT conversion studies undertaken. All peaks of interest (C=O, C-H and C-O) are highlighted, and show the same trends observed for the acrylate C=C stretch, i.e. minimal conversion during induction period, followed by rapid polymerisation and complete cure within 15 minutes.*

## X-ray Scattering

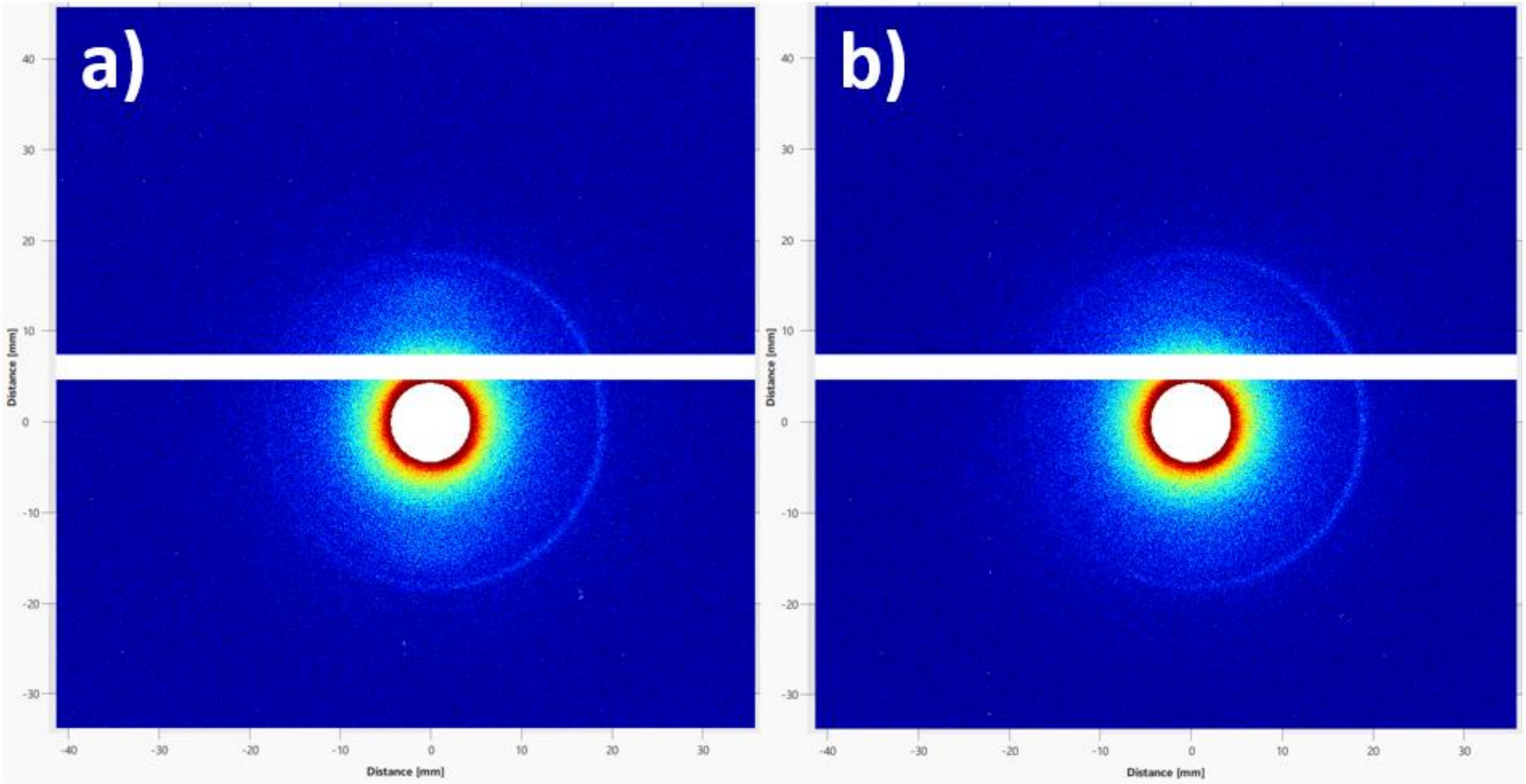


***Figure S11 –*** *An example of the small angle X-ray scattering data for a) the PET-RAFT LCE samples, and b) the free-radical LCE samples.*

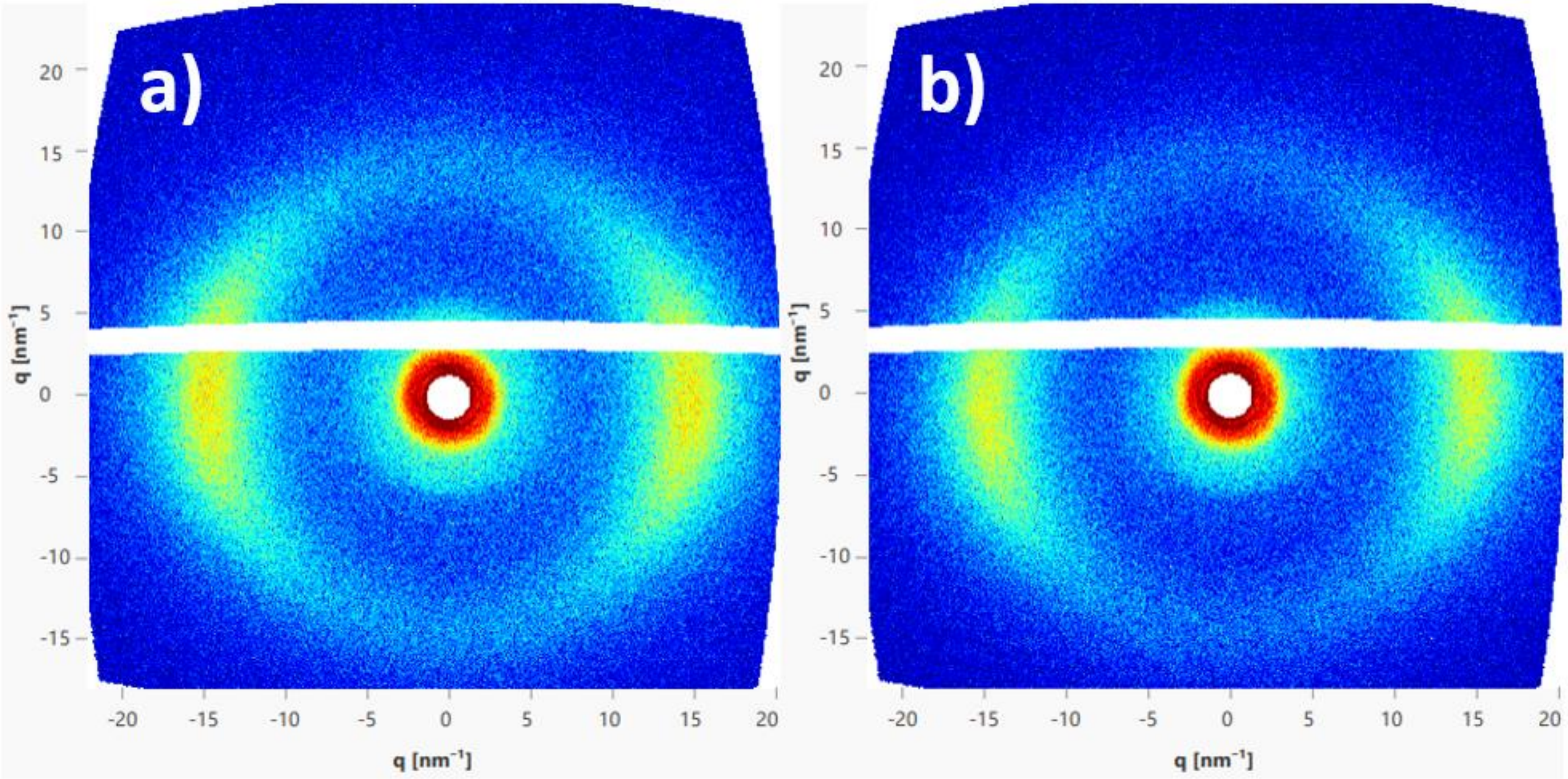


***Figure S12** – An example of the wide angle X-ray scattering data for a) the PET-RAFT LCE samples, and b) the free-radical LCE samples.*

## FTIR Analysis

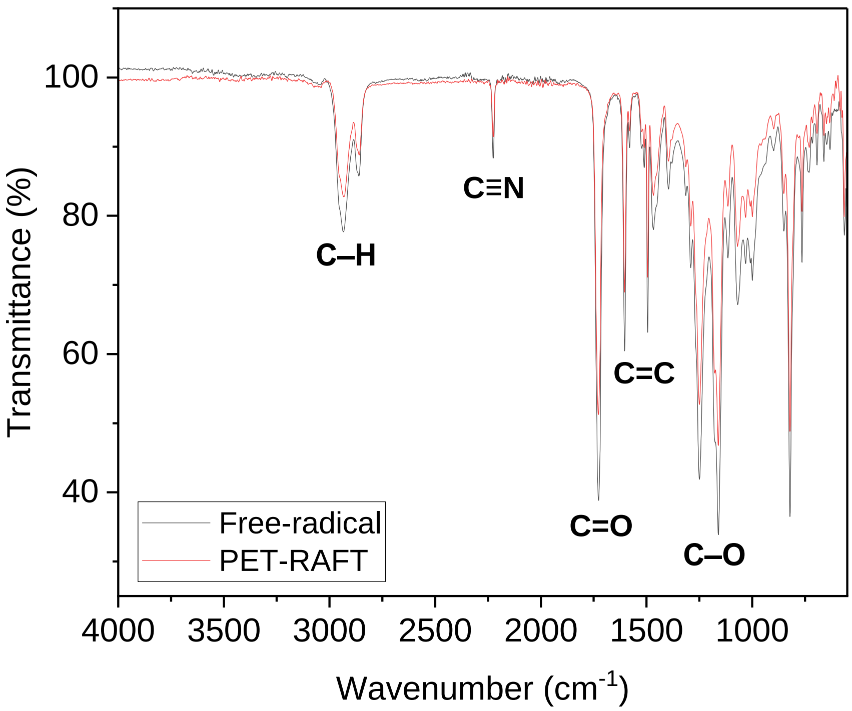


***Figure S13** - FTIR spectra comparison for the free-radical and PET-RAFT LCE samples.*

***Table S1** – Transmittance values obtained from normalised FTIR spectra for multiple positions in a PET-RAFT LCE sample.*

| | | **Transmittance (%)** | | |
|---|---|---|---|---|
| **Wavenumber ($cm^{-1}$)** | **Bond** | **Position 1** | **Position 2** | **Position 3** |
| **2933** | C-H | 81.9 | 82.1 | 82.0 |
| **1728** | C=O | 50.8 | 50.8 | 49.8 |
| **1603** | C=C | 68.1 | 68.8 | 70.1 |
| **1495** | C=C | 69.7 | 70.6 | 72.2 |
| **1161** | C-O | 46.1 | 46.5 | 46.7 |
| **1068** | C=S | 74.6 | 75.1 | 75.6 |

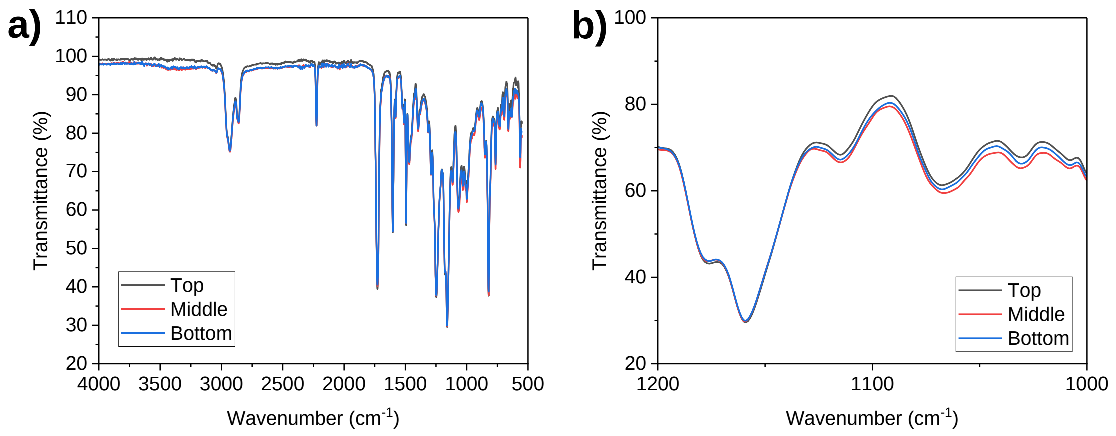


***Figure S14*** *– a) Overlaid FTIR spectra taken for both samples surfaces and the bulk material for an ~200 µm thick PET-RAFT elastomer sample normalized to the 2226 cm$^{-1}$ absorbance, and b) the region of 1200-1000 cm$^{-1}$ in the normalized FTIR spectra. The data demonstrates the homogeneity of the samples throughout the sample thickness.*

***Table S2*** *– Transmittance values obtained from normalised FTIR spectra for multiple positions in a PET-RAFT LCE sample.*

| | | **Transmittance (%)** | | |
|---|---|---|---|---|
| **Wavenumber (cm$^{-1}$)** | **Bond** | **Top** | **Middle** | **Bottom** |
| **2932** | C-H | 75.7 | 75.2 | 75.4 |
| **1728** | C=O | 39.4 | 40.3 | 40.6 |
| **1603** | C=C | 54.2 | 54.4 | 54.4 |
| **1494** | C=C | 56.0 | 56.5 | 56.6 |
| **1159** | C-O | 29.5 | 29.8 | 29.9 |
| **1068** | C=S | 61.3 | 59.6 | 60.3 |

## LCE Thickness Measurements

***Table S3*** *– Thickness measurements made at varying positions across the PET-RAFT LCE samples, displaying average values and the maximum percentage variation from the average value that was observed.*

| | **Thickness (µm)** | | | |
|---|---|---|---|---|
| **Measurement** | **Sample 1** | **Sample 2** | **Sample 3** | **Sample 4** |
| **1** | 241 | 272 | 292 | 280 |
| **2** | 250 | 277 | 298 | 289 |
| **3** | 252 | 275 | 303 | 285 |
| **4** | 255 | 263 | 309 | 267 |
| **5** | 256 | 276 | 300 | 286 |
| **Average** | 251 | 273 | 302 | 281 |
| **Variation (%)** | 4 | 4 | 3 | 5 |

***Table S4** – Thickness measurements made at varying positions across free-radical LCE samples, displaying average values and the maximum percentage variation from the average value that was observed.*

| Measurement | Thickness (µm) | | | |
|---|---|---|---|---|
| | **Sample 1** | **Sample 2** | **Sample 3** | **Sample 4** |
| **1** | 125 | 126 | 109 | 133 |
| **2** | 114 | 114 | 109 | 143 |
| **3** | 124 | 120 | 105 | 137 |
| **4** | 117 | 132 | 103 | 138 |
| **5** | 130 | 117 | 101 | 132 |
| **Average** | 122 | 122 | 105 | 137 |
| **Variation (%)** | 7 | 8 | 8 | 4 |

## References


1 T. Raistrick, Z. Zhang, D. Mistry, J. Mattsson and H. F. Gleeson, *Phys Rev Res*, 2021, **3**, 023191.

2 Z. Wang, T. Raistrick, A. Street, M. Reynolds, Y. Liu and H. F. Gleeson, *Materials*, 2023, **16**, 393.

3 D. Mistry, S. D. Connell, S. L. Mickthwaite, P. B. Morgan, J. H. Clamp and H. F. Gleeson, *Nat Commun*, 2018, **9**, 5095.

4 Y. Hayata, S. Nagano, Y. Takeoka and T. Seki, *ACS Macro Lett*, 2012, **1**, 1357–1361.
